# Gastrointestinal Interoception and Relapse in Anorexia Nervosa

**DOI:** 10.1001/jamapsychiatry.2026.1301

**Published:** 2026-06-17

**Authors:** Charles Verdonk, Keller Mink, Emily Choquette, Scott E. Moseman, Ahmad Mayeli, Jennifer L. Stewart, Martin P. Paulus, Ryan Smith, Sahib S. Khalsa

**Affiliations:** 1 Laureate Institute for Brain Research, Tulsa, Oklahoma; 2 UMR VIFASOM, Université de Paris, Paris, France; 3 French Armed Forces Biomedical Research Institute, Brétigny-sur-Orge, France; 4 Laureate Eating Disorders Program, Tulsa, Oklahoma; 5 University of Pittsburgh, Pittsburgh, Pennsylvania; 6 Oxley College of Health Sciences, University of Tulsa, Tulsa, Oklahoma; 7 Deputy Editor, *JAMA Psychiatry*; 8 Department of Psychiatry and Biobehavioral Sciences, Semel Institute for Neuroscience and Human Behavior, David Geffen School of Medicine, University of California, Los Angeles

## Abstract

**Importance:**

Anorexia nervosa (AN) is a deadly psychiatric disorder with relapse rates approaching 50% after weight restoration. Disrupted gastrointestinal interoception may underlie persistent symptoms and relapse vulnerability.

**Objective:**

To examine behavioral, computational, neural, and physiological markers of gastrointestinal interoception in weight-restored individuals with AN and test their association with relapse.

**Design, Setting, and Participants:**

This crossover trial was a single-blind, within-participant, randomized (block-order) trial conducted at the Laureate Institute for Brain Research between August 2021 and February 2025. Participants were females with weight-restored restrictive AN and age- and sex-matched healthy comparators (HCs). All participants ingested a vibrating capsule that delivered counterbalanced blocks of normal- and enhanced-intensity gut stimulation. Behavioral detection performance, electroencephalography, peripheral physiology, and computational modeling were used to assess interoception.

**Main Outcomes and Measures:**

Experimental-session measures included interoceptive accuracy, prior beliefs, interoceptive precision, learning rates, gastric-evoked potentials (GEPs), and hunger. Main clinical outcomes at 6 months included relapse status and symptom severity.

**Results:**

The cohort included 62 female participants with weight-restored restrictive AN (mean [SD] age, 18.9 [4.5] years) and 57 age- and sex-matched healthy comparators (HCs; mean [SD] age, 20.7 [5.3] years). Six-month follow-up data were collected for 54 participants with AN. Compared with HCs, participants with AN showed lower perceptual accuracy (Cohen *d* = −0.98; 95% CI, −1.51 to –0.44; *P* = .001) and higher miss rates (Cohen *d* = 1.02; 95% CI, 0.55 to 1.48; *P* < .001). Computational modeling revealed in the AN group stronger prior expectations that capsule vibrations would not be present (Cohen *d* = −0.31; 95% CI, −0.67 to 0.05; *P* = .05), greater shifts in interoceptive precision between blocks (Cohen *d* = 0.38; 95% CI, 0.02 to 0.75; *P* = .01), and learning asymmetries (vibration: Cohen *d* = −0.40; 95% CI, −0.77 to −0.04; *P* = .007; no-vibration: Cohen *d* = 0.35; 95% CI, −0.02 to 0.71; *P* = .01). GEP amplitudes did not differ by group but were correlated with accuracy and learning in AN. Capsule stimulation induced greater hunger increases in AN (interaction: η^2^p = 0.04, *P* = .04; AN: Cohen *d* = 0.94; 95% CI, 0.57 to 1.30; HCs: Cohen *d* = 0.40; 95% CI, 0.03 to 0.77). At follow-up, relapse was predicted by initial priors (odds ratio [OR], 3.82; 95% CI, 1.02 to 15.91; *P* = .05), response bias (OR, 5.37; 95% CI, 1.15 to 32.04; *P* = .04), and stomach unpleasantness (OR, 5.73; 95% CI, 1.38 to 33.5; *P* = .03), while eating disorder symptom severity was predicted by miss rate (β = 1.05; *R^2^* = 0.08; *P* = .05), difference in interoceptive precision (β = 5.84; *R^2^* = 0.16; *P* = .004), and initial priors (β = −2.99; *R^2^* = 0.09; *P* = .05).

**Conclusions and Relevance:**

In this study of weight-restored females with AN, gastrointestinal interoception was disrupted across multiple domains, including reduced accuracy detecting gut signals, maladaptive priors, rigid learning, and abnormal hunger rating. Several interoceptive markers predicted relapse and symptom severity at follow-up. These findings support the use of ingestible mechanosensory probes and computational modeling as scalable tools to monitor treatment response and guide relapse prevention in eating disorders.

**Trial Registration:**

ClinicalTrials.gov Identifier: NCT05111977

## Key Points

**Question:**

Is gastrointestinal interoception abnormal in anorexia nervosa, and do corresponding markers predict relapse and symptom severity at 6 months?

**Findings:**

In this crossover trial, female participants with anorexia nervosa showed abnormal gastrointestinal interoception at the behavioral and computational level, despite intact neural and physiological responses, compared with healthy participants. Behavioral and computational markers of disrupted gastrointestinal interoception predicted relapse and symptom severity.

**Meaning:**

This study provides evidence that disruptions of gastrointestinal interoception in anorexia nervosa predict relapse and may serve as scalable biomarkers to personalize treatment and prevent recurrence.

## Teaser

This crossover trial examines behavioral, computational, neural, and physiological markers of gastrointestinal interoception in weight-restored individuals with anorexia nervosa and tests their association with relapse.

## Introduction

Anorexia nervosa (AN) is a severe and often enduring psychiatric disorder with one of the highest premature mortality rates.^1,2^ Despite treatment, relapse rates are alarmingly elevated: up to 50% within 1 year of weight restoration.^3,4^ This persistent risk underscores the critical need to better understand the underlying pathophysiological mechanisms and for objective biomarkers that can track treatment response and predict long-term outcomes.

Interoception, the nervous system’s process of sensing and interpreting signals from within the body,^5^ has been consistently implicated in the symptomatology of AN^6,7^ and is often regarded as a candidate mechanism underlying AN pathophysiology.^8,9^ Empirical work has implicated abnormal interoception (particularly cardiac and respiratory) in appetite dysregulation, anxiety, and distorted body image.^10,11,12^ However, much less is known about gastrointestinal (GI) interoception in AN despite its central role in satiety, meal anticipation, and visceral discomfort.^13^ Methodological limitations have hampered GI-focused research, with most tools being either invasive (eg, insertion of inflatable balloons) or poorly suited for repeated clinical use (eg, water loading tests).^14^

To address this gap, we previously validated a minimally invasive approach involving an ingestible vibrating capsule delivering controlled mechanosensory stimulation to the stomach and adjacent gut segments.^15^ In healthy individuals, vibrating capsules reliably evoked nonaversive gut sensations and a neural signature measurable via electroencephalography (EEG), including parieto-occipital event-related potentials (ie, gastric-evoked potentials; GEPs) that correlated with perceptual accuracy.^15^ We further incorporated a bayesian computational model of interoceptive inference to estimate latent perceptual processes (internal computations not directly observable but inferred from behavior), including initial prior beliefs (the starting value of the prior probability over vibration vs no-vibration states, reflecting baseline expectations about vibration frequency), interoceptive precision (the reliability assigned to gut sensory evidence when inferring the capsule’s state), and learning (the rate at which prior beliefs are updated across trials). In this framework, prior beliefs reflect expectations about bodily states before sensory evidence is observed, whereas interoceptive precision reflects the degree to which incoming visceral signals are weighted when updating those expectations. For example, an individual with AN who expects that eating will produce painful fullness (a strong prior belief) may interpret mild gastric sensory signals as strongly aversive if those signals are weighted weakly relative to the expectation, whereas higher interoceptive precision would allow incoming sensory evidence to update or override that expectation. Previous work shows that interoceptive precision relates primarily to parieto-occipital EEG responses, whereas learning-related parameters relate to frontal EEG responses.^16^

Computational accounts of AN suggest symptoms result from aberrant interoceptive inference, characterized by a mismatch between anticipated bodily states (ie, prior beliefs) and incoming sensory input.^17,18,19^ This mismatch may involve overweighted priors and/or underweighted sensory input, with directionality varying across interoceptive channels and contexts.^13^ Neuroimaging evidence supports this view, showing exaggerated insula responses during anticipation but reduced responses during respiratory stimulation, consistent with stronger priors and reduced sensory precision.^20^ Moreover, it has been proposed that biased learning may help maintain these maladaptive expectations, potentially explaining persistence of disordered eating behaviors after weight normalization and broader rigidity in updating internal-state beliefs.^17,18,19^

In the current study, we tested whether inpatient individuals with weight-restored AN exhibit disrupted GI interoception across behavioral, computational, neural, and physiological domains (see trial protocol in Supplement 1). We predicted that relative to healthy comparators (HCs), individuals with AN would show reduced interoceptive accuracy (ie, a model-free behavioral measure of perceptual accuracy derived from hit and false-alarm rates), diminished GEP responses to vibratory gut stimulation, stronger prior beliefs against detecting capsule vibrations, lower interoceptive precision (a model-derived latent computational parameter reflecting reduced reliability assigned to incoming gut sensory evidence), and impaired learning. Further, we hypothesized that these parameters would predict relapse status and symptom severity 6 months after discharge, identifying potential biomarkers of prognosis and treatment response.

## Methods

The study was approved by the WCG institutional review board. All participants provided written informed consent and were financially compensated. This study followed Consolidated Standards of Reporting Trials (CONSORT) reporting guideline.

### Participants

Participants included weight-restored females with restrictive AN and sex- and age-matched HCs, recruited from the Laureate Eating Disorder Program inpatient unit and the surrounding community, respectively.

AN inclusion criteria were (1) female sex; (2) age 13 to 40 years, encompassing the most common developmental window for AN onset and persistence^21,22^; (3) body mass index (BMI) 18.5 or greater (to minimize starvation confounds^23,24^); and (4) primary restrictive AN diagnosis confirmed by the treating psychiatrist. Comorbid generalized anxiety disorder, specific phobia, dysthymia, or major depressive disorder were permitted because of their high prevalence in AN.^25^ Stable psychiatric medication use (>1 week) was allowed (eTable 1 in Supplement 2). Exclusion criteria included active suicidality, history of severe purging, pregnancy/lactation, or serious GI illness (eTable 2 in Supplement 2).

Healthy comparators were screened using the MINI interview^26^ to rule out *DSM-5* diagnoses.

### Experimental Session

The protocol comprised a 30-minute resting baseline, followed by 2 counterbalanced blocks (normal, enhanced) of vibratory stimulations delivered by a swallowed ingestible capsule (Vibrant Ltd) (eFigure 1 in Supplement 2). Participants were randomized to the order of stimulation blocks, not to group assignment or capsule condition. Stimulations began 3 minutes after ingestion, with each block consisting of approximately 60 stimulations of 3 seconds’ duration.^15^ Participants pressed and held a button on detecting capsule-induced sensations and released it once the sensation ceased (eMethods 2 in Supplement 2). A digital stethoscope affixed to the abdomen verified vibration timing^15^ (eMethods 2 in Supplement 2).

### Follow-Up

Participants with AN were followed up remotely at 1, 3, and 6 months postexperiment. This report focused on 6-month relapse and symptom outcomes, which provide the greatest sensitivity for detecting full relapse using standardized definitions.^3,4^

### Measures

#### Behavioral and Self-Report Measures

Interoceptive accuracy was quantified via normalized A-prime (eMethods 3 in Supplement 2).^27,28^ Response bias (−1 to +1) captured button press tendencies (eMethods 3 in Supplement 2). Additional metrics included miss rate and response time (latency from vibration onset to button press).

Before and after the task, participants completed visual analog scale ratings assessing intensity and valence of stomach/digestive, breath, and heartbeat sensations; muscle tension (intensity only); and current hunger, thirst, and urges to urinate/defecate (eMethods 3 in Supplement 2).

#### Electroencephalographic Measures

Scalp electroencephalographic (EEG) activity was recorded using a 32-channel cap (10-20 system; Brain Products GmBH), with 31 EEG channels and 1 electrocardiogram channel sampled at 1000 Hz.

Preprocessing used EEGLAB (toolbox version 19.0^29^), and custom Matlab 2021a scripts (MathWorks). Data were downsampled (250 Hz), notch filtered (60 Hz), bandpass filtered (0.1 Hz to 80 Hz), and cleaned via Independent Component Analysis to remove eyeblinks and saccadic eye movements, muscle activity, and motion-related artifacts. Artifact independent components were identified using decision criteria from the aE-REMCOR^30^ and the rtICA^31^ approaches. Data were referenced to the mastoid electrodes (TP9/TP10), epoched around vibration onset and offset (−200 milliseconds to +3000 milliseconds), and baseline corrected (−200 milliseconds previbration mean).

#### Computational Modeling

Participant responses were used to fit a validated bayesian model of interoceptive inference developed for this task.^16^ Estimated parameters included: (1) interoceptive precision, reflecting the expected precision or reliability of the afferent interoceptive signal; (2) difference in interoceptive precision across blocks with differing vibration intensity (normal vs enhanced); (3) initial prior belief about the frequency of vibrations; and (4) learning rates for vibration and nonvibration trials, reflecting the degree to which prior expectations change based on whether vibrations are (or are not) presented within a given time frame. Parameters were estimated using a variational Bayes optimization algorithm (Variational Laplace).^32,33^ A detailed model description is available (eMethods 4 and eTables 3 and 4 in Supplement 2).

#### Peripheral Physiological Measures

Electrogastrogram, cardiac, and skin conductance signals were recorded during baseline and stimulation blocks (eMethods 5 in Supplement 2).

#### Definition of 6-Month AN Illness Status

Six-month AN illness outcomes were operationalized into relapse, remission, or partial recovery based on self-reported symptoms, behaviors, and BMI, following standardized criteria^4^ (eMethods 6 in Supplement 2).

### Statistical Analyses

Linear mixed-effects models were applied for behavioral, computational, physiological, and self-report data, with age and BMI as covariates. EEG was analyzed using cluster-based permutation tests. Multilevel associations were computed using Spearman correlations conducted separately within each group, with mean GEP amplitude calculated within the spatiotemporal window identified as the common neural marker of gut mechanosensation across groups. In the AN group, logistic and linear regressions tested prediction of 6-month relapse outcomes or global scores on the Eating Disorder Examination Questionnaire (EDE-Q). Statistical significance was defined as *P* < .05 (2-tailed). Detailed analysis description available (eMethods 7 in Supplement 2).

## Results

**Figure 1.**
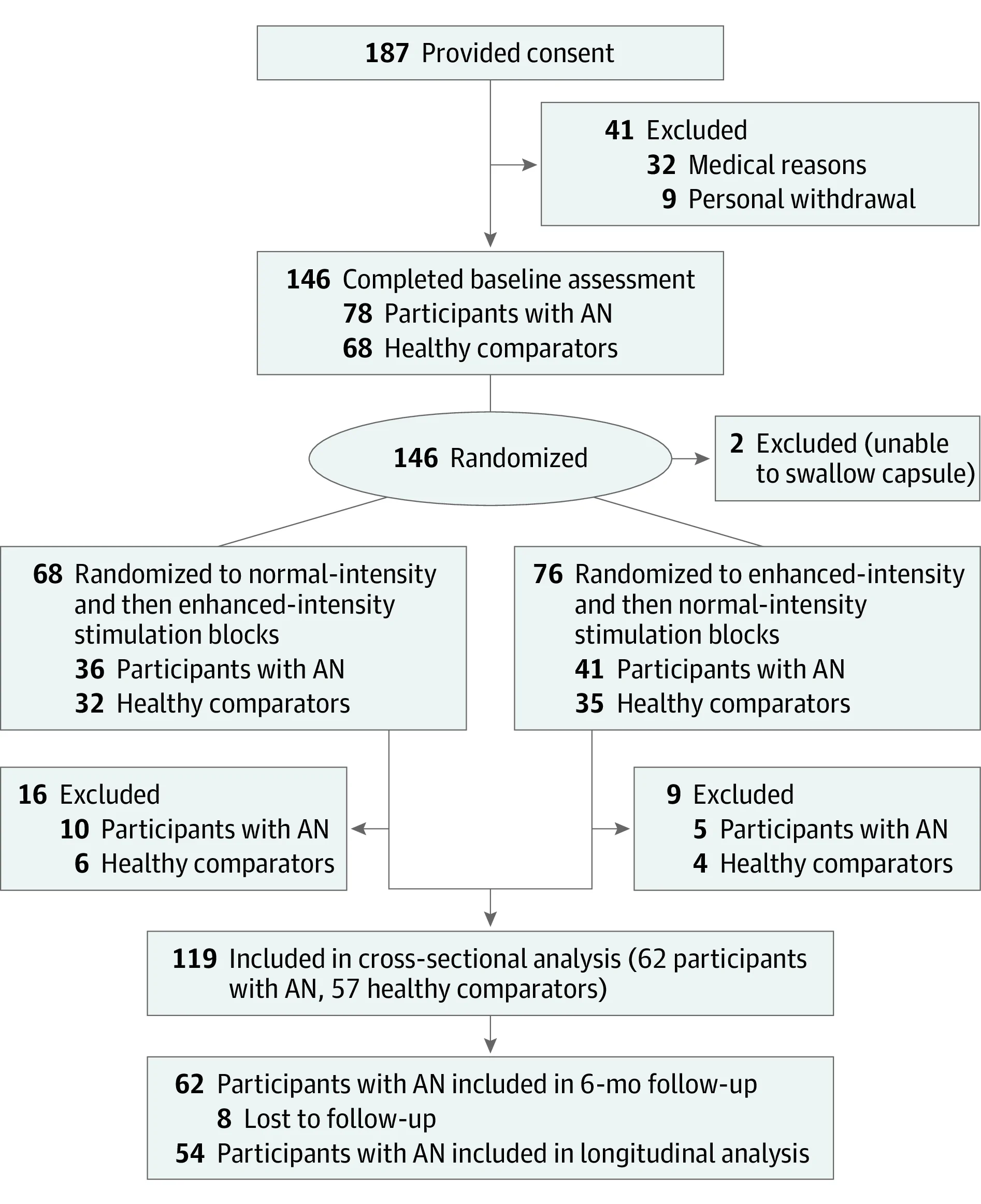
CONSORT Flow Diagram for the Study AN indicates anorexia nervosa.

For the cross-sectional analysis, 119 female participants with analyzable data were included, comprising 62 individuals with AN restricting subtype and 57 HCs (Table and Figure 1).

**Table.** Summary of Demographic and Clinical Information for Participants With AN and Healthy Comparators Included in the Cross-Sectional Analysis

Variable	Mean (SD)		*P* value	Log(BF10)
	Participants with AN-R (n = 62)	Healthy comparators (n = 57)		
Current age, y	18.9 (4.5)	20.7 (5.3)	.03	−0.14
Body mass index^a^				
At inpatient admission	16.9 (2.3)	NA	NA	NA
At experimental session	19.9 (1.7)	22.2 (2.9)	<.001	6.90
AN characteristics, y				
Age at onset	14.9 (2.9)	NA	NA	NA
Illness duration	3.9 (3.2)			
Comorbid psychiatric conditions, No. (%)		NA	NA	NA
Generalized anxiety disorder	43 (69)	NA	NA	NA
Major depressive disorder	23 (37)	NA	NA	NA
Obsessive compulsive disorder	12 (19)	NA	NA	NA
Psychiatric medication, No. (%)^b^	57 (92)	NA	NA	NA
EDE-Q total score at predischarge visit	3.79 (1.06)	0.46 (0.50)	<.001	12.44

Abbreviations: AN-R: anorexia nervosa, restricting subtype; EDE-Q, Eating Disorder Examination Questionnaire; log(BF10): log scale of Bayes factor 10; NA, not applicable.

^a^ Calculated as weight in kilograms divided by height in meters squared.

^b^ For a detailed list of psychiatric medications, refer to eTable 1 in Supplement 2.

### Behavioral Findings

**Figure 2.**
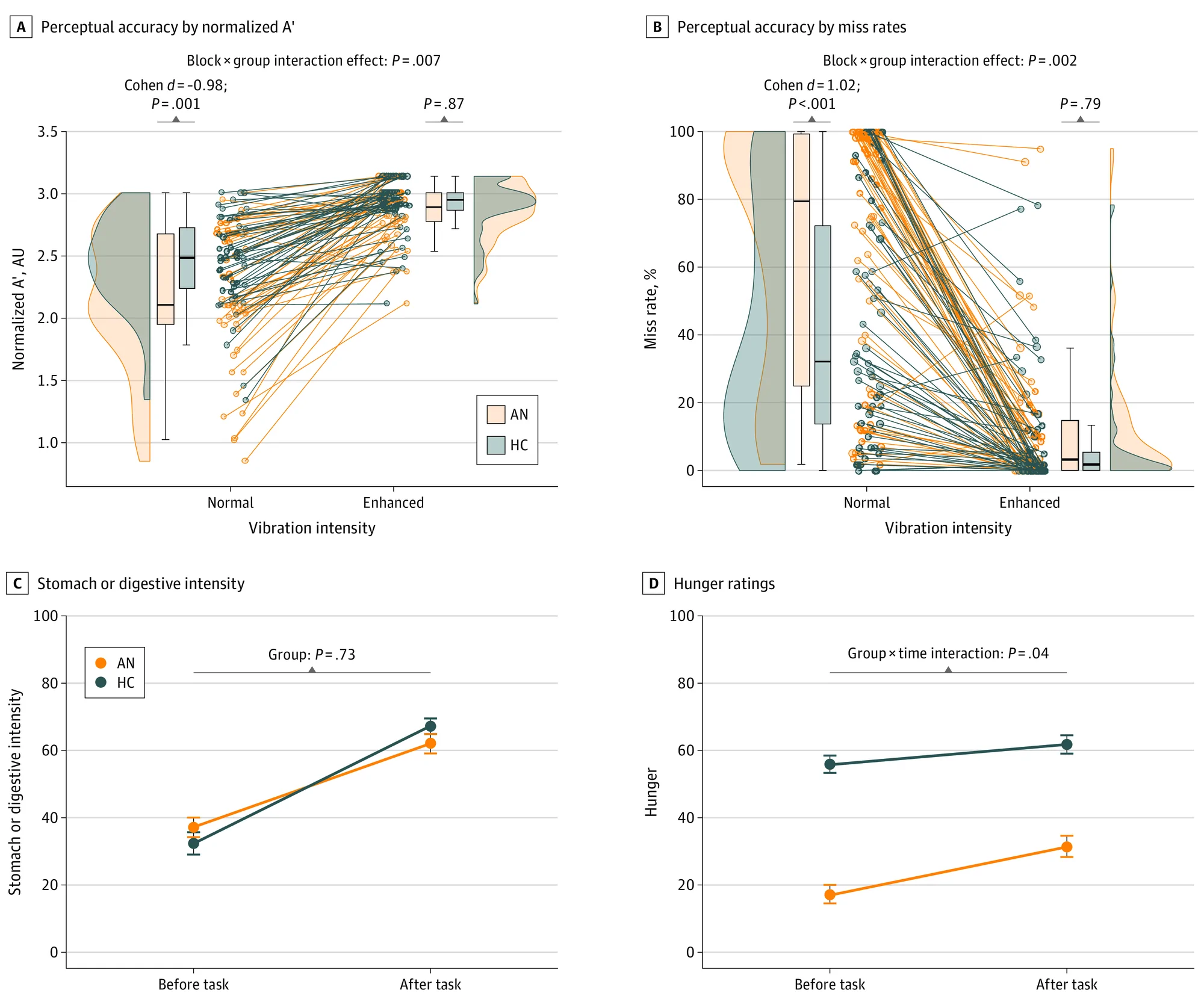
Plots of Behavioral and Self-Report Measures of Gastrointestinal Interoception Participants with anorexia nervosa (AN) exhibited significantly lower perceptual accuracy, as measured by lower normalized A-prime (A) and higher miss rates (B), in detecting normal but not enhanced stimulations compared with healthy comparators (HCs). C, Intensity of stomach/digestive sensations increased during the task in both groups (significant time effect) but with no group differences. D, Hunger ratings showed a group × time interaction, with larger task-associated increases in the AN group. Error bars indicate standard error of the mean.

Linear mixed-effects models showed significant block × group interactions for normalized A-prime (η^2^p = 0.07; *P* = .007) and miss rate (η^2^p = 0.08; *P* = .002). Post hoc comparisons revealed that, during the normal stimulation condition, participants with AN exhibited significantly lower perceptual accuracy, as indicated by lower normalized A-prime values (Cohen *d* = −0.98; 95% CI, −1.51 to −0.44; *P* = .001; Figure 2A) and higher miss rates (Cohen *d* = 1.02; 95% CI, 0.55 to 1.48; *P* < .001; Figure 2B) than HCs, whereas no group differences were observed during enhanced stimulation (normalized A-prime: *P* = .87; miss rate: *P* = .79). A group effect was also observed for response bias, with participants with AN less likely to press the button (η^2^p = 0.07, *P* = .02), while no group differences emerged for response times (*P* = .13). Additional block effects are described in eResults 1 in Supplement 2.

### Self-Report Findings

Stomach/digestive sensation intensity increased during the task (η^2^p = 0.49, *P* < .001), with no group differences (*P* = .73) (Figure 2C). Beyond stomach sensations, AN individuals reported higher breathing and muscle tension intensities and larger task-related increases in heartbeat intensity (eResults 1 and eFigure 2 in Supplement 2). Stomach/digestive unpleasantness showed a marginal group difference (*P* = .06) and no group × time interaction, suggesting similar task-evoked changes across groups (eResults 1 in Supplement 2). Regarding homeostatic urges, hunger showed a group × time interaction (η^2^p = 0.04, *P* = .04; Figure 2D), with post hoc comparisons indicating that task-induced increases were larger in individuals with AN (Cohen *d* = 0.94; 95% CI, 0.57-1.30; *P* < .001) than in HCs (Cohen *d* = 0.40; 95% CI, 0.03-0.77; *P* = .03). Thirst decreased from pretask to posttask in both groups and was lower in AN than in HCs, whereas urges to urinate and defecate increased from pretask to posttask in both groups and were higher in AN vs HCs (eResults 1 in Supplement 2).

### Electroencephalogram Findings

**Figure 3.**
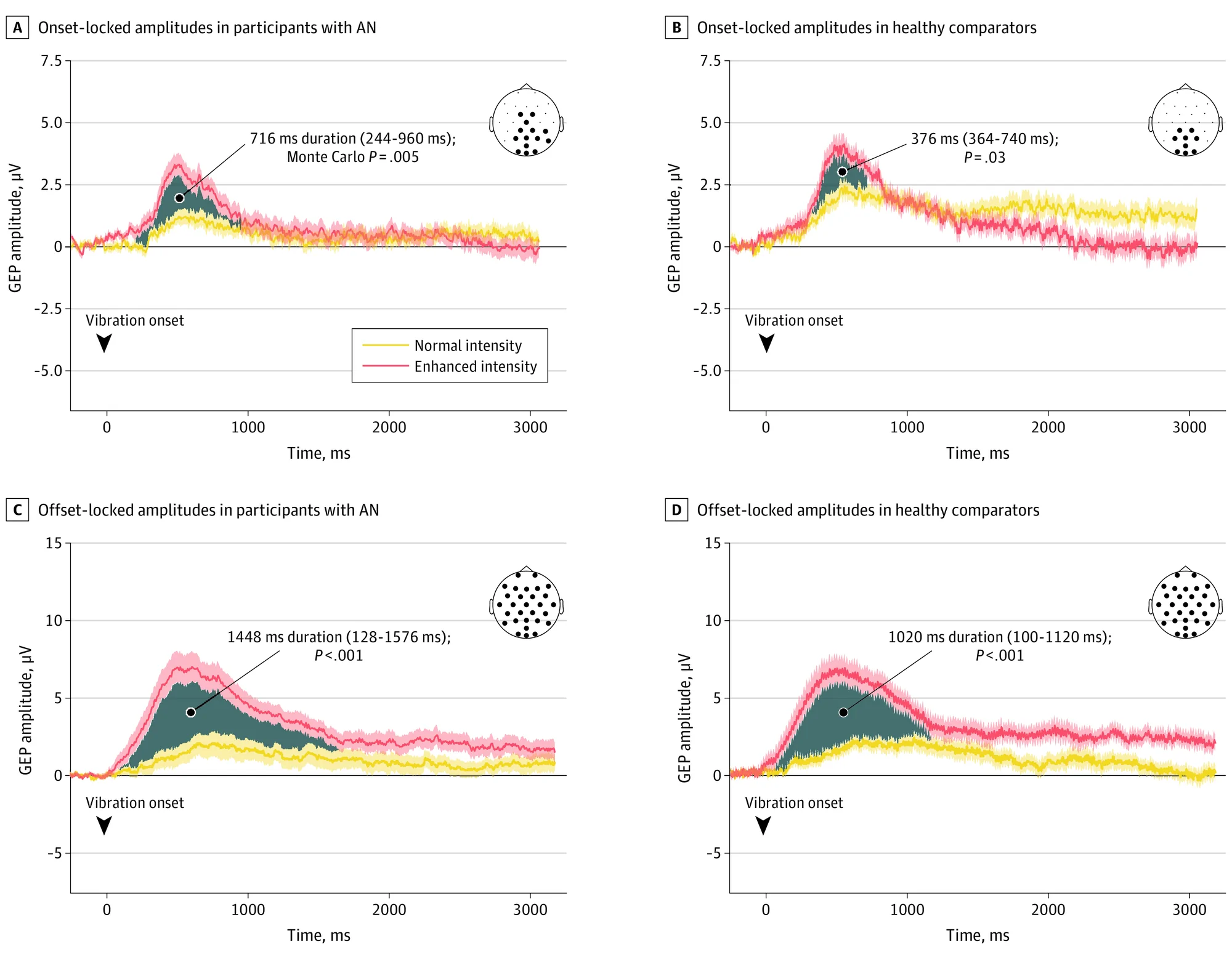
Plots of Gastric-Evoked Potentials (GEPs) During Vibratory Mechanosensory Gut Stimulation Enhanced-intensity stimulations elicited significantly larger GEP amplitudes compared with normal-intensity stimulations in both groups. A, In the anorexia nervosa (AN) group, onset-locked GEP amplitudes were significantly greater during enhanced-intensity stimulation (Cohen *d* = 0.54; 95% CI, 0.27-0.80), with effects observed in centroright parieto-occipital electrodes. B, In healthy comparators, onset-locked GEP amplitudes were also significantly greater during enhanced-intensity stimulation (Cohen *d* = 0.53; 95% CI, 0.25-0.81), with a partially overlapping set of parieto-occipital electrodes. C, In the AN group, offset-locked GEP amplitudes were significantly larger during enhanced stimulation across all electrodes. D, In healthy comparators, offset-locked GEP amplitudes were likewise significantly larger during enhanced stimulation, with effects spanning all electrodes. The gray shaded area highlights the temporal window where a significant difference was observed between normal- and enhanced-intensity vibrations. Error bars indicate standard error of the mean.

Cluster-based permutation testing showed no significant group differences in vibration-onset GEP amplitude in either block(eFigure 3 in Supplement 2). However, enhanced stimulations elicited significantly larger GEP amplitudes compared with normal stimulation in both groups. In the AN group, this effect occurred within a 716-millisecond window, from 244 milliseconds to 960 milliseconds after vibration onset (Cohen *d* = 0.54; 95% CI, 0.27-0.80; Monte Carlo *P* = .005), involving the following electrodes: P3, P4, O1, O2, P8, Cz, Pz, Oz, FC1, FC2, CP1, CP2, CP6, and POz (Figure 3A). In HCs, the effect was observed within a shorter (376-millisecond) window, from 364 milliseconds to 740 milliseconds after the vibration onset (Cohen *d* = 0.53; 95% CI, 0.25-0.81; Monte Carlo *P* = .03), involving a similar but not identical set of electrodes: P3, P4, O1, O2, Pz, Oz, CP1, CP2, and POz (Figure 3B). Similar results were observed for vibration-offset GEP analyses (eResults 2 and eFigure 4 in Supplement 2). In the AN group, enhanced stimulation elicited significantly greater GEP amplitudes than normal stimulation across all electrodes, within a 1448-millisecond window from 128 milliseconds to 1576 milliseconds postoffset (Monte Carlo *P* < .001; Cohen *d* = 0.63; 95% CI, 0.36-0.90) (Figure 3C). In HCs, the same effect was observed from 100 milliseconds to 1120 milliseconds postoffset, also across all electrodes (Monte Carlo *P* = .002; Cohen *d* = 0.71; 95% CI, 0.42-0.99) (Figure 3D).

### Computational Findings

**Figure 4.**
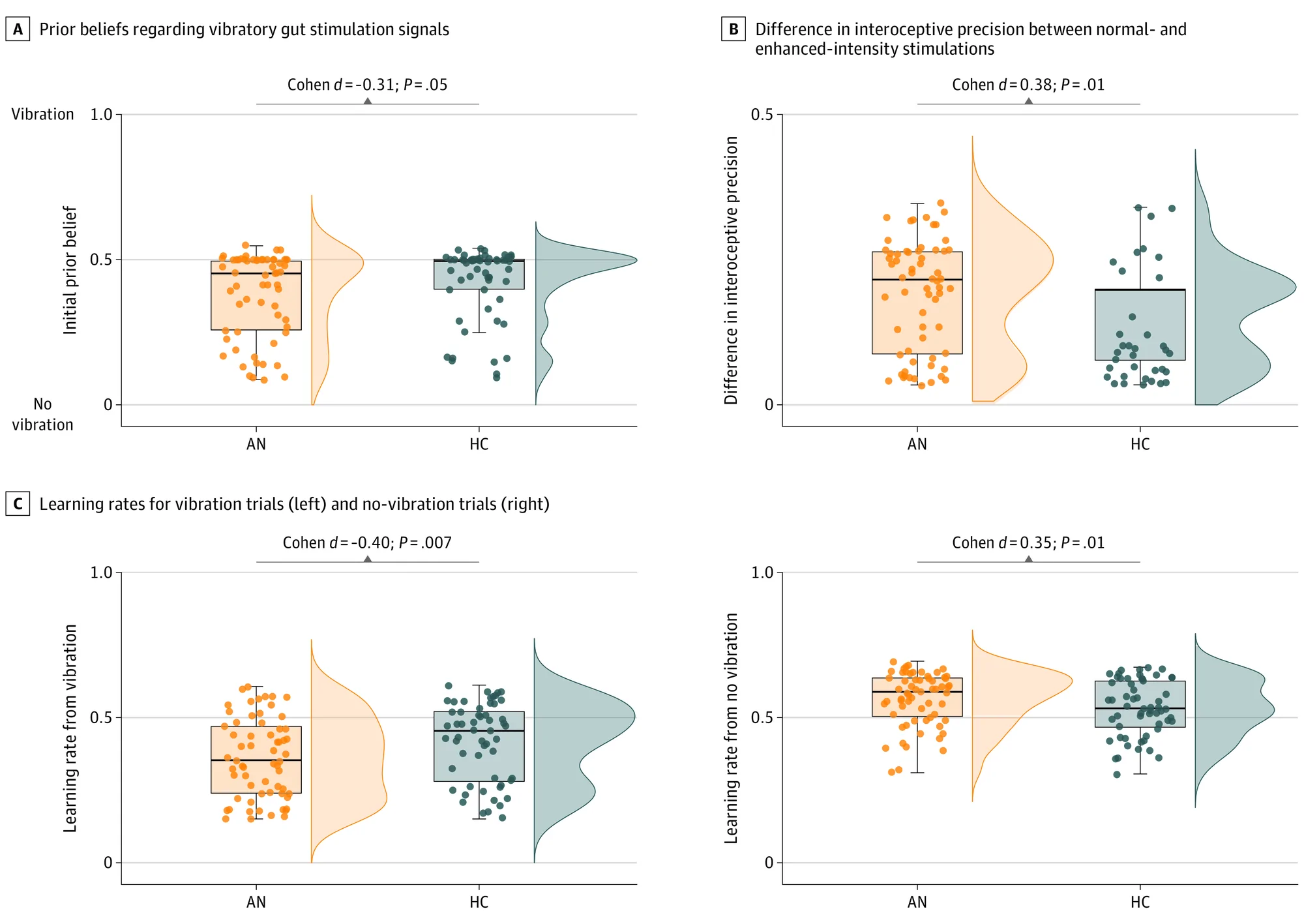
Computational Measures of Gastrointestinal Interoception Compared with healthy comparators (HCs), the anorexia nervosa (AN) group exhibited stronger initial prior beliefs against perceiving vibratory gut stimulation signals (A), a larger difference in interoceptive precision between normal- and enhanced-intensity stimulations (B), and significantly different learning rates from both vibration and no-vibration trials (C). The plots display the distribution of individual data points, corresponding boxplots, and individual participant values.

Compared with HCs, the AN group exhibited initial prior beliefs that more strongly favored the absence of perceiving GI signals, as indicated by prior belief values further below the neutral value of 0.5 (Cohen *d* = −0.31; 95% CI, −0.67 to 0.05; *P* = .05) (Figure 4A). The AN group also exhibited a greater difference in interoceptive precision between the normal and enhanced stimulation blocks (Cohen *d* = 0.38; 95% CI, 0.02 to 0.75; *P* = .01; Figure 4B), where higher values indicate greater increases in perceptual precision under stronger stimulation, but there was no significant group difference in overall interoceptive precision. Finally, the AN group showed slower learning rates than HCs for trials with vibrations (Cohen *d* = −0.40; 95% CI, −0.77 to −0.04; *P* = .007) and faster learning rates than HCs for trials without vibrations (Cohen *d* = 0.35; 95% CI, −0.02 to 0.71; *P* = .01) (Figure 4C). Model selection/recoverability details and exploratory visualization of prior time-course differences are available (eResults 3, eTables 5 and 6, and eFigure 5 in Supplement 2).

### Longitudinal Findings

At 6 months (n = 54 individuals with AN with follow-up; 16 in full relapse; Figure 1 and eResults 4 and eFigure 6 in Supplement 2), relapse was successfully predicted by experimental session measures, including initial prior beliefs (odds ratio [OR], 3.82; 95% CI, 1.02-15.91; *P* = .05) (eTable 7 in Supplement 2), response bias in the normal block (OR, 5.37; 95% CI, 1.15-32.04; *P* = .04), and self-reported stomach/digestive unpleasantness (assessed using pretask and posttask visual analog scale ratings; OR, 5.73; 95% CI, 1.38-3.35; *P* = .03) (eTable 8 in Supplement 2). Eating disorder symptom severity at 6 months, indexed by EDE-Q scores, was predicted by miss rate in the normal block (β = 1.05; *P* = .05; adjusted *R^2^* = 0.08), difference in interoceptive precision between normal and enhanced stimulations (β = 5.84; *P* = .004; *R^2^* = 0.16), initial prior beliefs (β = −2.99; *P* = .05; *R^2^* = 0.09), pretask stomach/digestive intensity (β = 0.02; *P* = .01; *R^2^* = 0.13), and stomach/digestive unpleasantness before (β = −0.03; *P* = .02; *R^2^* = 0.11) and during the task (β = −0.04; *P* = .02; *R^2^* = 0.11) (eResults 4 and eTables 9 and 10 in Supplement 2). Positive regression coefficients [β >0] indicate that higher predictor values were associated with greater symptom severity at follow-up, whereas negative coefficients indicate the opposite.

Peripheral physiological findings and multilevel correlational analyses are presented in eResults 5 and 6, respectively, in Supplement 2.

## Discussion

In this study, we applied a clinically scalable mechanosensory stimulation paradigm to probe GI interoception in weight-restored inpatient females with restrictive AN, revealing numerous disruptions across behavioral and computational levels. Relative to matched healthy comparators, individuals with AN showed reduced accuracy in detecting normal-intensity gut sensations. Computational modeling revealed that participants with AN held stronger prior beliefs that capsule-induced gut vibrations would not be present and maladaptive learning from vibratory stimulations. Although GEP amplitudes did not differ overall between groups, individuals with AN showed earlier and more prolonged differences between conditions, along with stronger correlations between neural signals and behavioral/computational measures during normal-intensity stimulation. Notably, several task-derived markers and self-report ratings predicted clinical relapse and symptom severity at 6 months, identifying them as candidate prognostic biomarkers. Lastly, the AN group exhibited a larger posttask increase in hunger ratings, suggesting an altered homeostatic response to gut stimulation.

These findings offer direct empirical evidence for disrupted GI interoception in weight-restored individuals with AN, reflected by reduced interoceptive accuracy in detecting mechanosensory gut signals. They advance theoretical models implicating interoceptive dysfunction as a core mechanism in AN pathophysiology^14^ and address a critical translational gap by shifting the focus of inquiry from cardiac and respiratory signals to gastrointestinal interoception, a modality central to feeding behavior and symptom expression.^13,34^ Prior studies of GI interoception in AN have been limited by methodological barriers, with most available tools being invasive or poorly suited for repeated trials necessary to derive psychophysiological or computational phenotypes.^14^ This addition is important as it provides specificity for the type of limitation of the water load task being referenced (which can only deliver one symptom measure across the trial timepoint).

The vibrating capsule approach overcomes these limitations, offering a noninvasive, repeatable, and developmentally appropriate method for probing gut-brain signaling across adolescence and young adulthood, when AN often emerges or is maintained.^21,22^

Computational modeling revealed altered interoceptive inference in AN, characterized by stronger prior expectation that capsule-induced gut vibrations would not be present and by maladaptive learning dynamics. Within a bayesian framework, this pattern reflects an increased expectation that the vibratory stimulation is unlikely to occur or will not be perceived. These findings align with bayesian computational models of interoceptive dysfunction, which suggest that abnormally high confidence in prior expectations (ie, hyperprecise priors) can override conflicting sensory input, thereby reducing perceptual updating and contributing to persistently distorted bodily perception in psychiatric disorders.^17,19^ In AN, overly strong (ie, hyperprecise) priors may downweight ascending visceral signals, contributing to misinterpreted or blunted satiety cues. This mechanism may also apply to hunger and thirst signals, facilitating the ability of individuals with AN to ignore these bodily cues.^35^ Supporting this possibility, AN participants showed an asymmetric learning pattern in which they updated their beliefs more in response to the absence of gut sensations than their presence. This pattern led to a growing expectation that vibrations would not occur, even when they did. This dynamic parallels known cognitive inflexibility in AN^36^ and suggests a mechanistic link between perceptual inference and executive dysfunction. Future studies should investigate whether this rigidity reflects interoceptive-specific learning or general impairments in inhibitory cognitive control.^37^ More broadly, our findings extend prior computational work in the visual and cardiac domains^38,39^ highlighting that disruptions in interoceptive inference persist after weight restoration. These results underscore recent clarion calls to move beyond nutritional rehabilitation alone,^40^ and suggest that computational interoceptive markers could inform the development or augmentation of novel brain-gut therapies in AN by focusing on recalibrating maladaptive priors and promoting flexible learning from visceral input.^41^

From a neurophysiological perspective, this study provides the first evidence to our knowledge linking GEPs to behavioral and computational markers of GI interoception in AN. Although overall amplitudes did not differ by group, clear onset- and offset-locked responses emerged across participants, with amplitudes strongly modulated by stimulation intensity. These results underscore that vibratory gut stimulation reliably engages cortical activity involved in visceral signal processing, highlighting its value as a sensitive tool for probing gut-brain communication. Importantly, GEP amplitudes during normal-intensity stimulation were closely associated with perceptual accuracy and learning rates, particularly in the AN group. This suggests that GEPs may reflect both bottom-up sensitivity to mechanosensory input and top-down flexibility in belief updating. Beyond amplitude, individuals with AN also showed earlier onset and prolonged duration of GEP responses. Although the functional significance of this expanded temporal envelope remains unclear, it may reflect heightened salience attribution or sustained attentional engagement with visceral stimuli, as suggested by previous studies on event-related potential duration.^42,43^ Such prolonged engagement could contribute to persistent monitoring of bodily signals in AN, reinforcing symptom expression and potentially increasing relapse risk.

Longitudinal analyses revealed that behavioral and computational interoceptive markers, as well as self-reported stomach sensations, predicted relapse in AN. Notably, stomach/digestive unpleasantness during the task predicted outcome despite only marginal group differences at baseline, suggesting that even subtle subjective differences (ie, undetectable via standard group comparisons) may carry prognostic value. This underscores the utility of ecologically relevant, task-based measures that go beyond resting-state or self-report (ie, questionnaire-based) assessments, which may be compromised in AN by limited introspective ability or alexithymia.^44^

Intriguingly, while stomach unpleasantness may signal heightened visceral threat sensitivity, the larger increase in hunger after mechanosensory stimulation among individuals with AN*—*despite lower baseline ratings and identical pretask fasting*—*suggests that suppressed hunger signaling can be re-engaged through vibratory gut stimulation, potentially by increasing the salience of afferent gastric signals and allowing sensory evidence to exert a stronger influence on interoceptive inference. This observation raises the possibility that controlled capsule-based gastric mechanosensory stimulation may help restore homeostatic interoceptive function and reconnect patients with internal hunger cues. However, although hunger increased during the task, we did not observe corresponding changes in the valence of stomach sensations and did not assess the emotional or behavioral interpretation of hunger itself. Thus, it remains unclear whether enhanced hunger recognition would promote adaptive eating behavior or interact with cognitive factors that maintain restrictive responses. If replicated, such bottom-up stimulation paradigms could help support meal initiation and augment refeeding efforts during early recovery. Together, these findings support the use of interoceptive perturbation tasks not only for stratifying relapse risk, but also for identifying novel targets to enhance treatment engagement in AN.

Self-report ratings also revealed modality-specific alterations in AN. Perceived stomach sensation intensity increased with stimulation but did not differ by group, and self-reported stomach unpleasantness showed only marginal group differences, despite robust behavioral (ie, task) impairments in AN. This dissociation between objective and subjective measures highlights the limitations of relying solely on subjective ratings to capture visceral processing abnormalities and underscores the need for concurrent behavioral and computational assessment. Notably, the substantial interindividual variability (especially within the AN group) suggests there is meaningful heterogeneity in GI interoceptive processing, which may have implications for personalized risk stratification and treatment targeting. For example, future studies could examine whether individuals with the greatest interoceptive impairment are most likely to benefit from interoceptive feedback training or targeted gut-focused interventions. For additional discussion of peripheral physiological findings and their interpretation, as well as potential implications of the behavioral findings for interoceptive training and personalized interventions, see the eDiscussion in Supplement 2.

### Limitations

This study has several limitations, including the female-only restrictive AN sample, single-site recruitment, diagnoses established during routine clinical care rather than structured interviews, and the relatively short follow-up period (eDiscussion in Supplement 2).

Despite the limitations, this study demonstrated that weight-restored females with AN exhibit marked disruptions in gastrointestinal interoception, reflected in reduced perceptual accuracy for gut mechanosensory signals, stronger expectations of not feeling gut sensations, and biased belief updating that progressively reinforces these expectations over repeated input. These abnormalities were evident across behavioral and computational domains, and several markers predicted relapse and symptom severity, highlighting their potential as clinically actionable biomarkers. Clinically, these interoceptive disruptions may compromise the ability to detect and interpret hunger and fullness cues, prompting greater reliance on rigid cognitive rules around eating. Such reliance may hinder treatment response, particularly during refeeding, and heighten relapse risk if bodily beliefs remain inflexible despite changing internal signals. By integrating a noninvasive ingestible capsule with computational modeling and EEG, this study establishes a scalable framework for probing and tracking gut-brain function in eating disorders. More broadly, the findings support personalized approaches to treatment stratification and the development of brain-gut interventions targeting recalibration of bodily beliefs and restoration of appetite awareness in AN.

## Conclusions

In this study of weight-restored females with AN, gastrointestinal interoception was disrupted across multiple domains. These findings support the use of ingestible mechanosensory probes and computational modeling as scalable tools to monitor treatment response and guide relapse prevention in eating disorders.

## Notes

Supplement 1. Trial protocol

Supplement 2. **eMethods 1. **Participants **eTable 1. **Diagnostic comorbidities, psychotropic medication status, and race of anorexia nervosa (AN) and healthy comparison (HC) participants **eTable 2. **Exclusion criteria **eMethods 2. **Experimental protocol **eFigure 1.** Experimental setup **eMethods 3. **Behavioral and self-report measures **eMethods 4. **Computational modeling **eTable 3.** Prior means for key model parameters **eTable 4.** Overview of model variants and their corresponding parameters **eMethods 5. **Processing of peripheral physiological data **eMethods 6. **Illness status definitions **eMethods 7. **Statistical analysis **eResults 1. **Behavioral and self-report findings **eFigure 2**. Retrospective ratings of interoceptive sensations and homeostatic urges before, during, and after vibratory gut stimulation **eResults 2. **Electroencephalogram findings **eFigure 3. **Group overlay of onset-evoked gastric-evoked potential (GEP) responses **eFigure 4. **Group overlay of offset-evoked gastric-evoked potential (GEP) responses **eResults 3. **Computational findings **eTable 5.** Model comparison results **eTable 6.** Correlation results for parameter recoverability analysis **eFigure 5.** Prior time-course **eResults 4. **Longitudinal findings **eFigure 6. **Individual trajectories of eating disorder symptoms from discharge to 6-month follow-up in AN **eTable 7.** Logistic regression results predicting 6-month AN status (full relapse) from experimental session measures **eTable 8. **Self-report measures: logistic regression predicting 6-month AN illness status (full relapse) from retrospective ratings of sensation intensity and valence (unpleasant/pleasant) and state items before, during, and after vibratory gut stimulation **eTable 9.** Linear regression results predicting EDE-Q Total score at 6 months from experimental session measures **eTable 10.** Self-report measures: linear regression results predicting EDE-Q Total score at 6 months from retrospective ratings of sensation intensity and valence (unpleasant/pleasant) and state items before, during, and after vibratory gut stimulation **eResults 5. **Peripheral physiological findings **eFigure 7. **Peripheral physiological responses during baseline and vibratory gut stimulation **eResults 6. **Multilevel correlation findings **eTable 11.** Correlations between average gastric-evoked potential (avGEP) amplitude and behavioral or computational measures for the normal and enhanced stimulation blocks
eDiscussion

eReferences

Supplement 3. Data sharing statement
