# Supplementary material for: Gastrointestinal Interoception and Relapse in Anorexia Nervosa
Source: JAMA Psychiatry. 2026 Jun 17:e261301. Online ahead of print. doi: 10.1001/jamapsychiatry.2026.1301 (PMC13276667; doi:10.1001/jamapsychiatry.2026.1301)
Supplement: Supplement 1. — Trial protocol [file jamapsychiatry-e261301-s001.pdf]

LIBR # 2021-007
07/25/2024

Title of Project: A neurocomputational assay of gastrointestinal interoception in anorexia nervosa

Principal Investigator: Sahib Khalsa, M.D., Ph.D.

Address:
Laureate Institute for Brain Research
6655 S Yale Ave
Tulsa, OK 74136-3326

Phone:
918-502-5743

Fax:
918-502-5135

Email:
skhalsa@laureateinstitute.org

Sponsors:
National Institute of Mental Health and Laureate Institute for Brain Research

A. Purpose of the study

Given that anorexia nervosa (AN) has the highest mortality rate of any psychiatric illness and current treatments show limited efficacy, there is a crucial need to better understand the brain mechanisms driving the pathophysiology of this disorder. This proposal combines an experimental medicine approach focused on gastrointestinal (GI) interoception with computational modeling to probe neural circuits of interoception and appetite-related gastric processing in AN. The goal is to identify perceptual and neural markers for AN at the individual patient level and apply statistical methods to evaluate clinical outcomes longitudinally. Supported by our preliminary data, this proposal is based on the premise that the pathophysiology of AN includes a computational dysfunction manifested by cognitive suppression of the expected precision of afferent interoceptive signals associated with hunger, which reduces their motivational influence and facilitates maladaptive and avoidant eating behaviors. We propose a case-control study with AN and healthy comparisons who will undergo baseline testing using a novel GI interoception probe during measurement of symptoms, behavior, and neural and physiological responses. Sensory stimulation will occur during the premeal period, anchoring responses to an anticipatory context with high relevance to the disorder. These individuals will be followed for up to 6 months to examine clinical outcomes. Clinical outcomes measured include events such as Hospitalization, Serious illness, Therapy, and other life events (e.g., accidents, changes in medication status). A computational approach will examine the basic hypothesis that AN individuals have lower sensory precision for GI interoception and that the degree of sensory imprecision is related to clinical characteristics. Moreover, we will examine the relationship of this imprecision to neural and physiological responses. We will then apply regression-based approaches to these neurophysiological and perceptual measures to longitudinally test associations with clinical outcomes. Additional activities include targeted assessment of the physical location of gastric stimulation, and non-invasive evaluation of gastric motility. Achieving the aims of this project will provide unique insights into the pathophysiology of AN by arbitrating whether AN is a consequence of “top-down” or “bottom-up” dysregulation in the nervous system, which could transform our understanding of how intrinsic interoceptive disturbances lead to AN. Pragmatically, it will result in new technologies for identifying interoceptive dysfunction at the individual level, allowing psychiatry to develop diagnostic and predictive biomarkers of AN. Thus the neurocomputational assay of gastrointestinal interoception in AN could be used to develop low-cost, scalable, and objective tools for identifying dysfunction in individual patients, to facilitate neurobiologically-based definitions of recovery, and to predict the risk of relapse following treatment. Finally, this proposal lays the groundwork for the future development of precision psychiatric interventions such as perceptual retraining therapies to target (and recalibrate) abnormal brain- body interactions.

Specific Aim 1: To determine the perceptual and EEG-related signatures of dysregulated computational processing during GI interoception in female inpatients with AN (ages 13-40 years, BMI 18.5 and above)

Hypothesis 1a: During stimulation of stomach sensations with a vibrating capsule, females with AN will exhibit attenuated interoceptive sensory precision estimates (IP), a concomitantly stronger influence of prior expectations (pGI), and a lower learning rate (η, following from reduced attention to interoceptive signals), relative to age- and sex-matched healthy comparisons (HC).

Hypothesis 1b: During stimulation of stomach sensations with a vibrating capsule, females with AN will show a reduced EEG evoked response potential from the brain relative to matched HCs, but no difference in gastric myoelectric (EGG) responses – a pattern consistent with a top-down suppression of interoceptive signals.

Aim 2: To determine the clinical relevance of expectation-based interoceptive dysregulation in AN

Hypothesis 2a: Computational and neurophysiological individual difference measures collected during the stimulation of stomach sensations with a vibrating capsule (pGI, IP, η, event related potential, ERP amplitude) will accurately predict post-discharge outcomes (relapse or remission) in AN at 1, 3, and 6 months. Lower IP and η, stronger influences of pGI, and lower ERP amplitude will be predictive of poorer outcomes.

Hypothesis 2b: Computational and neurophysiological measures will be associated with measures of AN illness severity (lowest BMI, current eating disorder severity).

B. Background and Significance

Anorexia nervosa (AN) has one of the highest mortality rates of any psychiatric illness (Arcelus et al., 2011), yet the pathophysiology of this disorder is poorly understood and current treatments show limited efficacy. Abnormalities of interoception, the brain-body connection, have been suggested to play a central role in manifestation of AN (Khalsa et al., 2018a), partly because of the phenotype of severe food restriction, ignoring of hunger signals, and extremely low body weight that characterize the disorder. However, few studies have rigorously examined the influence of interoception on illness expression in AN.

In our previous studies, we have demonstrated that individuals with AN show abnormal subjective awareness of interoceptive signals. For example, using the adrenaline analogue isoproterenol we found that individuals with AN report feeling cardiorespiratory sensations with greater intensity when anticipating eating a meal (Khalsa et al., 2015), an effect that was particularly pronounced for sensations of dyspnea. In a follow up study, we found that these patients were also more inaccurate at localizing cardiovascular sensations in the body, suggesting an impairment in the ability to accurately discriminate interoceptive signals occurring within the body (Khalsa et al., 2018b). These abnormalities are accompanied by evidence of abnormal activation in the insular cortex, a key hub for interoceptive processing in the brain (Hassanpour et al., 2018). For example, other studies have shown that current AN patients demonstrate greater insula activation than HC when attending to heartbeat sensations and lower insula activation to stomach sensations (Kerr et al., 2016), and recovered AN patients show experience greater choking sensations and show greater insula activation during an inspiratory breathing load paradigm (Berner et al., 2017).

Despite evidence of abnormal cardiorespiratory perception in AN, it appears that the primary abnormality of heightened visceral perception pertains to the gastrointestinal system. AN patients show a nearly superhuman ability to voluntarily ignore hunger signals, and prolonged severe food restriction clearly impacts the state of the gastrointestinal system. In clinical settings, AN patients commonly report gastrointestinal complaints such as exaggerated fullness in response to small meals (i.e., postprandial fullness), early satiety, abdominal pain, and they also complain of bladder and bowel symptoms outside of meal times, such as fullness, bloating, and constipation (Robinson,

1989, Halmi and Sunday, 1991, Sato and Fukudo, 2015).The physiology underlying these abnormal perceptions has not been extensively studied in research settings. Of the studies that are available, most have used a naturalistic study design following inpatients during the refeeding process. For example, acutely ill inpatients with AN report symptoms of premature fullness after eating small amounts of food (Peterson et al., 2016, Bluemel et al., 2017, Heruc et al., 2018). Further suggesting a heightened visceral sensitivity is the finding that fructose-sorbitol ingestion disproportionately provokes gastrointestinal symptoms in inpatients with eating disorders. After short-term refeeding to promote weight gain and restore homeostatic balance to the gut AN patients continue to report exaggerated fullness (Peterson et al., 2016), although to a somewhat lower extent (Bluemel et al., 2017). These symptoms appear to decrease substantially in the 6 months following inpatient treatment (Salvioli et al., 2013), raising the possibility that they reflect a marker or indicator of successful treatment response.

In a previous WIRB approved project (WIRB # 20190971) we developed a novel probe for measuring perceptions of the gastrointestinal system using a minimally invasive approach, via ingestion of a vibrating capsule (Vibrant, Ltd.) in healthy individuals and inpatients with AN. In the current study we aim to recruit another sample of inpatients with AN to mechanistically determine the relationship between stimulation of gastrointestinal perceptions, eating disorder symptoms, neural response (via electroencephalography or EEG), and longitudinal treatment response. We additionally aim to determine the physical location of capsule stimulation and perform non-invasive evaluations of gastric motility in HC and AN. This systematic program of research has the potential to substantially improve our understanding of the pathophysiological basis of anorexia nervosa by directly clarifying the link between gastrointestinal signaling and elevated reports of symptoms arising from within it. In the long term, it could lead to new clinical tests capable of differentiating individual risk prognosis (i.e., biomarkers for treatment response prediction), and new treatments based on recalibrating or retraining faulty perceptions of the body (i.e., development of new exposure therapies for helping eating disorder patients reducing elevated interoceptive stress levels surrounding meal times).

C. Preliminary Studies and Description of Laboratory:

Investigator background: The principal investigator of this protocol, Sahib Khalsa MD, PhD, is a board-certified psychiatrist. A primary focus in the laboratory has been the development and testing of novel physiological probes to better understand the role of interoception in eating disorders. For example, in previous work approved by WIRB he has adapted an isoproterenol infusion protocol to measure interoception in patients with eating disorders and anxiety disorders during functional magnetic resonance imaging (fMRI) scanning (e.g., WIRB # 1155298). As mentioned previously, another protocol (WIRB # 20190971) involved initial development of the vibrating capsule approach. Dr. Khalsa has a robust publication record demonstrating the development and adaptation of methods for studying interoception within psychiatric patient samples, in premier neuroscience and psychiatric journals. Of the additional investigators on the protocol (Martin Paulus MD, Scott Moseman MD, Ryan Smith PhD), two are board-certified licensed psychiatrists (Martin Paulus MD, Scott Moseman MD medical director of Laureate Eating Disorders Program) who can assist the principal investigator in the event that any adverse psychological effects should arise during the course of the study. Additionally, Martin Paulus, MD serves as the on-site co-principal investigator to provide local oversight of study procedures, personnel, and participant safety, given that Dr.

Khalsa is now based at a different institution (University of California, Los Angeles). Dr. Khalsa continues to serve as the principal investigator and primary study contact, and actively supervises the study remotely in collaboration with the on-site study team.

Rationale for Vibrating capsule approach: Most prior studies attempting to study the conscious perception of gastrointestinal sensations have used invasive approaches. These involve insertion of an inflatable balloon into the esophagus (Aziz et al., 1997, Hobson et al., 2000), stomach (Salet et al., 1998), colon (Holzl et al., 1996), or rectum (Hobday et al., 2000). While balloon distension approaches have shown the ability to engage putative interoceptive cortical neural circuitry (i.e. insular and somatosensory cortices) (Aziz et al., 2000a, Aziz et al., 2000b, Naliboff et al., 2006), the invasiveness of these approaches and reliance upon a Gastroenterologist consultant for placement is severely limiting to mental health practice settings. Other less invasive approaches exist but these too have certain limitations. For example, a water loading test involves the ad-libitum ingestion of water until reaching a feeling of fullness (Herbert et al., 2012); while masking prevents participants from deriving external clues suggesting fullness, this procedure can only be repeated once per testing session, providing limited information about the perceptual processes underlying gastrointestinal sensation, and no information related to the processing of solid food. Other approaches have involved delivery of small amounts of tastants directly onto the tongue, such as sucrose solution (Frank et al., 2008, Wagner et al., 2008), or milk shakes (Stice et al., 2008, Bohon et al., 2009). While these approaches have clear validity as food sources, they engage a broad set of neural circuitry related to taste (e.g. orbitofrontal cortex, striatum, as well as insula) and they do not directly target the gastrointestinal perceptual processes in the stomach or colon. Prior investigators have attempted delivery of nutritive substances directly into the stomach in AN and observed abnormalities of perception (Coddington and Bruch, 1970), although these were in an extremely small AN sample (n=3) and again required an invasive approach. While each of these approaches has utility in generating insights into the pathophysiology of interoception in AN, none of them have yielded a clinical test capable of providing and independent marker of illness severity or predictor of treatment response. The current approach seeks to accomplish the first steps towards this objective via development of a minimally invasive measure of gastrointestinal perception.

Preliminary studies: The Vibrant capsule was developed by Vibrant Ltd, with the intention of providing a non-pharmacologic therapeutic option for chronic constipation. It consists of an orally administered non-biodegradable vibrating capsule, that is wirelessly activated using an activation base unit. An initial clinical trial study examined the safety of this approach first in canines, and secondarily in healthy human volunteers (Ron et al., 2015) (clinicaltrials.gov: NCT01306448). After no adverse events were noted in these participants, as part of the same trial an open label safety study was conducted in 26 patient with chronic constipation, who received the Vibrant capsule twice a week for 7.5 weeks. There were no serious adverse events, all capsules were expelled without difficulty, and any minor adverse events (e.g. abdominal pain, diarrhea, flatulence) were transient. Subsequent to this study, a larger prospective randomized multi-center placebo controlled clinical trial testing the efficacy of the Vibrant capsule for treatment of chronic idiopathic constipation has been initiated (see WIRB® Protocol #20190204). The Vibrant capsule has been designated by the US Food and Drug Administration as a 501K De-Novo regulatory path (i.e., non-significant risk device).

During the previous WIRB protocol (WIRB # 20190971), we adapted the Vibrant

capsule to deliver mechanosensory stimulation to the stomach, and we have demonstrated the safety of this procedure in 40 healthy (21 male, 19 female) and 5 AN females, all with BMIs above 18.5 and ages within 13 to 40 years (our current proposed criteria is between ages 15 to 40 years). There were no adverse events or severe adverse events in any group. We observed no evidence of nausea, dizziness, or pain (0 out of 40 HC, 0 out of 13 AN). We have seen no evidence of increased anxiety on the State-Trait Anxiety Inventory (STAI) in HCs (pre STAI mean 27, post STAI mean 28) or ANs (pre STAI mean 46, mean post STAI 44). Further, across several inpatient focus groups the majority (>70%) of AN patients (n=25) expressed more favorable attitudes toward an ingestible capsule approach versus other cardiac, respiratory, or invasive GI methods.

Laboratory description: The Khalsa laboratory is housed within the Laureate Institute for Brain Research LIBR), a nonprofit research institute dedicated to the study of the neuroscientific basis of psychiatric disorders. The major research tools that LIBR researchers bring to bear in conducting this research has involved multimodal brain neuroimaging, genetics, novel therapeutics, and bioinformatics technologies. The Scientific Director and President is Martin Paulus, M.D. The LIBR faculty currently consists of eight full-time scientists who serve as independent principal investigators. They are joined by a team of more than 40 multidisciplinary scientists and clinical research and administrative staff from a range of backgrounds, including cognitive neuroscience, psychiatry, physics, psychology, neuroscience, computer science, genetics, research grants administration, and ethics oversight. The institute is staffed by four research dedicated registered nursing staff (RNs). The LIBR facility, located in Tulsa, Oklahoma is in the same building as the Laureate Eating Disorders Program, which has 33 inpatient beds, and provides over 2300 outpatient visits per year. LIBR is on the campus of the Laureate Psychiatric Clinic and Hospital (LPCH), which is part of the Saint Francis Health System (SFHS). LIBR has a formal affiliation with SFHS and LPCH aimed at facilitating access to patients with psychiatric disorders who are interested in participating in research protocols.

D. Research Design and Methods:

Overall protocol design

The experimental setup involves ingestion of the Vibrant capsule and vibratory stimulation of mechanosensors in the stomach. All participants will be asked to ingest a capsule. They will be informed that they may receive a capsule that vibrates at some point or a placebo capsule that does not vibrate, but that neither they nor the experimenter will know whether any stimulations will occur. However, in actuality, every participant will receive a capsule that delivers vibratory stimulations at some point, making this a single blinded protocol. The stomach is the gastrointestinal target. Less than two minutes after ingestion participants will be asked to attend to the sensations coming from their stomach, and press a handheld button each time they feel a sensation that they ascribe to the capsule. They will begin receiving stimulations 2.5 minutes after ingestion. A block of 60 stimulations (normal intensity, 3 second duration) will be delivered in random order during a 12.5 minute period. After a 2.5 minute pause, a second block of 60 stimulations (enhanced vibration intensity, 3 second duration) will be

delivered during a 12.5 minute period (in counterbalanced order). Thus, participants will be asked to detect stomach sensations during a 30 minute period following capsule ingestion. This timing ensures the capsule is still in the stomach: the normal gastric emptying time is estimated to be ~30 minutes, and weight restored AN individuals show similar if not slightly delayed gastric emptying times (Diamanti et al., 2003, Benini et al., 2004, Bluemel et al., 2017)). Mode A entails a normal vibration amplitude (as developed by Vibrant) which minimizes perceptual detection, whereas Mode B entails an increased vibration amplitude which is expected to increase perceptual detection.

Participants will be monitored by a trained member of the study team present in the room throughout the entire stimulation session duration. A clinically trained member of the team (RN or MD) will always be available in the building during the stimulations in case additional participant monitoring is needed

Protocol summary:

a. Participants will rate their experience of gastrointestinal sensations via button presses starting immediately after capsule ingestion, and associated emotional responses.
b. Number of stimulation visits: 1 or 2 targeting stimulation of empty stomach.
c. Number of gastric mapping visits: 1
d. Participants enrolled: AN and healthy individuals

Once a participant successfully meets the screening inclusion and exclusion criteria they will participate in a face-to-face medical screening visit to verify eligibility for the study.

Recruitment
We plan to obtain measurements from 115 adult and teen inpatients with AN and 115 adult and teen healthy comparison participants over 5 years (see ‘Human Participants’ section for details). Participants will be recruited through a pre-approved LIBR Screening protocol (WIRB # 20200161, formerly 20101611), through the LIBR database, through flyers and social media advertisements, and through word of mouth via eating disorder clinicians at the Laureate Eating Disorders Program and in the community. In addition, teens will be recruited through local school districts using Peachjar (an online platform to deliver flyers to parents with information about afterschool activities).

Screening assessment:
After referral to this study by a clinician, AN patients will complete a brief face-to-face visit with a researcher. Then they will be consented into the study. Healthy comparisons will complete a brief phone screen and will be consented into the study. Once a participant signs the informed consent, they will start the study. Before completing any stimulation sessions, they will also be required to successfully meet the inclusion and exclusion criteria.

Self-report assessments:
Self-report measures may include demographics, measures of psychiatric health and eating disorder scales (see **Time and Events Table**).

NIMH Data Archive (NDA) Common Data Elements:

We are required to collect responses from participants to certain measures considered to be “common data elements” by the funding agency support this research (National Institute of Mental Health (NIMH) to submit to their NIMH Data Archive (NDA) as part of the funding agreement. These measures include the DSM-5 crosscutting assessment for adults, youth, and parents/guardians of youth; the WHODAS 2.0 and GAD-7 for adult participants; and the RCADS-25 for youth and parents/caregivers.

Psychiatric and Medical History:
All participants consenting to study will be asked to sign an authorization to release medical records from Laureate Psychiatric Clinic and Hospital.

General Procedure
Prior to starting the study, the experimenter will review the entire study procedure and informed consent document with the participant. Participants may be asked to complete a series of self-report questionnaires (see Time and Events Table) that will take between 1.5-2 hours to complete. These same measures may also be completed at the end of the study and during the follow up visit.

Psychiatric and Medical Evaluations:
Participants consenting to study entry may be asked a series of standardized questions about psychiatric or medical symptoms they have experienced during their lifetime, including a detailed medication history and family history. An unstructured psychiatric interview and a Structured Clinical Interview for DSM-IV Disorders (via the M.I.N.I. International Neuropsychiatric Interview (Sheehan et al., 1998)) may be administered as part of the determination of eligibility. These assessments will be obtained through the LIBR Screening protocol. All participants will undergo a physical exam including measurement of vital signs, taking a breathalyzer test, and submission of urine sample for pregnancy and drug screening. During this visit many of the self report assessments may be completed. This visit may be conducted over a period of up to 14 days.

Sensation and emotion measurement:
During the stimulation session participants will enter perceptions via button press in a custom application time locked to the capsule activation using the base activation unit. Before and after the stimulation session they will be given a smartphone/tablet preloaded with a custom smartphone application (‘Somatomap’). They will be taught how to provide ratings in Somatomap, including indicating areas of body concern, body sensation ratings, associated emotions, and influence on body size estimation. They will enter these ratings before and after the stimulation period. Additionally, they will enter these ratings during the 3-hour postprandial period of the High-density EGG assessment.

The primary outcome variables evaluated for this intervention will be changes in gastrointestinal perception during the different stimulation periods. Secondary outcome variables will be severity of eating disorder (indexed via the Eating Disorders Examination Questionnaire, EDE-Q), level of anxiety (State-Trait Anxiety Inventory, STAI, Anxiety Sensitivity Index 3, ASI-3), and depression (Patient Health Questionnaire, PHQ-9, and Inventory of Depressive and Anxiety Symptoms, IDAS-II).

Stop Signal and Tone Tasks
To measure behavioral and neural responses to inhibitory processing, participants will

complete the stop signal task (Matthews et al, 2005). At the onset of each trial, either an 'X' or an 'O' appears on a black background. Participants are instructed to press, as quickly as possible, the left button when an 'X' appeared, and the right button when an 'O' appeared. They are also instructed not to press either button whenever they hear a tone during a trial (stop trials). Each trial lasts approximately 1300 ms and each trial is separated by 200-ms interstimulus intervals (blank screen). Individual response latency is used to denote the period of inhibitory processing and provide a subject-dependent jittered reference function. Participants perform stop and nonstop trials, with a greater ratio of nonstop trials. Trial order is pseudo-randomized throughout the task and counterbalanced. Participants first perform the stop task in a training session in order to determine their mean reaction time (RT) from 'X' and 'O' stimuli onset. Such individual measures are used to determine the stop signal delay (SSD) for the sto p trials. The task takes 15 minutes in total.

The tone task is a control condition in which participants press a button in response to tones heard via computer speakers. This takes approximately five minutes.

High-density EGG assessment of stomach activity
We will use the Alimetry device to perform high density mapping of gastric motility. Briefly, this entails a 64 channel EGG assessment of gastric motility (Gharibans et al., 2017, Gharibans et al., 2018) following consumption of a small meal. Participants will arrive on an empty stomach and will then consume a liquid milkshake meal (containing approximately 600 kCal). For the next three hours they will lay recumbent on a couch will periodically providing ratings of gastric sensation including feelings of fullness/bloating, hunger, and satiety. During this period, participants will also enter sensation and emotion ratings via the Somatomap application. A major advantage of this device is the ability to obtain measurements of gastric movement without resorting to imaging methods such as fluoroscopy, which involve exposure to much higher amounts of radiation than abdominal X-rays (e.g. 6 mSV which is the equivalent of 2 years of natural background radiation).

Follow up:
Follow up will be collected remotely at three time points (1month, 3 months, 6 months) by having the participants complete rating scales in Redcap while in their home environment. Participants will be requested to complete these scales within 14 days of receiving them; however, responses uploaded outside of this time frame will also be accepted.

Physiological measurement during testing:
Heart rate (ECG), respiration, skin conductance, pulse oximetry, electroencephalography (EEG), and electrogastrogram will be recorded continuously using BIOPAC instrumentation (Goleta, California) or EEG (Brain Products GmBH, Germany) during each stimulation session. EEG is a non-invasive measure of brain function that is compatible with the Vibrant Capsule. During the second capsule stimulation visit continuous ECG, EEG, and EGG will not be recorded; however, we will record vital signs before and after stimulation as noted in the next section.

Safety monitoring during testing
Physiological vital signs will be recorded during the screening enrollment visit by a trained member of the study team present in the room. Following this initial visit, these

vital signs will be closely monitored by nursing staff and physicians on the inpatient unit. The study team will have full access to all vital sign data. Participants will be closely monitored for signs of discomfort or increased anxiety. Individuals reporting increased anxiety levels at any time during the assessment will be offered the opportunity to discontinue the study. Any participant displaying abnormally high elevations in pulse range (e.g. HR >120 bpm) or blood pressure (e.g. Systolic BP > 160, or Diastolic BP >100) will be excused from the study. During stimulations participants will be monitored by a trained member of the study team. A clinically trained member of the team (RN or MD) will always be available in the building during the stimulations in case additional participant monitoring is needed. In the event of emergency, the first responder alerting procedures will be followed, as specified in the emergency protocol section (below).

E. Statistical Methods:

Data will be analyzed using standard univariate and multivariate statistical parametric methods except for instances that require the use of non-parametric statistical analysis. Examples of these methods include linear mixed-effects models with Group (or stimulation condition) as the fixed-effect, and (a) random intercept only (equivalent to exchangeable correlation structure) or (b) random time/stimulation slope to capture temporal correlation at the 0.05 significance level (i.e., group by time effects). Additional statistical measures such as ANCOVAs, t-tests, and correlation analyses may be undertaken. There are no previous studies that have used this stimulation protocol (as it has not yet been developed), to assist in estimating expected effect sizes. Since there have been no prior analogous mechanosensory assays of stomach interoception in AN, we based our target sample size on the estimated proportional difference in relapse rates without treatment over the 6 month period following discharge (Aim 2a), taking into account estimated relapse rates for AN from the literature and possible expression of eating psychopathology in HC. Our prior literature review (Khalsa et al, 2017, J Eat Dis) suggested lifetime relapse rates between 30-50%, with the highest risk occurring within the first year after inpatient discharge. **Taking a conservative approach (equivalent to a medium effect size), we estimated a relapse probability of 25% within the AN sample** and an expression rate of 5% within the HC sample over the course of the study period. Detecting differences between these two independent proportions (95% power (1-beta error probability) at an alpha error probability of 0.05, G*Power 3.1231) yielded a total sample size of 130 subjects (n=65 completers per group; note that we aim to recruit 115 participants per group to account for an estimated 20% dropout rate across all study phases). During data processing, analysts will remain blinded to group membership whenever possible.

F. Gender/Minority/Pediatric Inclusion for Research

All efforts will be made to ensure that our participant population closely resembles the ethnic and racial composition of the greater Tulsa area. Women and minorities will be included in the study without prejudice according to their representation in the study population. Adult subjects will be recruited from the greater Metro Tulsa area and should thus share the racial and ethnic composition of this area. Adolescents aged 15 and up will be included in the study. We include adolescents for these reasons: 1) several forms of brain maturation occur during adolescence. Primary among them in females is the onset of menorrhea. Menstrual cycle onset requires hypothalamic-pituitary-adrenal

(HPA) axis engagement of ovarian and uterine responses and is complete in the general US population by age 15. 2) While the mean age at onset of AN is 18.5 +/- 5.1 years, there is substantial variability and age at onset has continued to decrease (Favaro et al, 2009 *J Clin Psychiatry*). Excluding AN adolescents would therefore miss a substantial portion of the age at onset for the AN population. 3) Prefrontal myelinization is an additional brain maturation phase but since this is not complete until the late 20s, we did not feel this developmental phase of prefrontal cortex maturation was an acceptable decision criterion for inclusion.

Time and Events Table for Study

Procedures	Screening Enrollment Visit 1	Visit 2 (e.g. empty stomach)	Visit 3 (Gastric Mapping)	1-month Follow-Up Visit 4	3-month Follow-Up Visit 5[c]	6-month Follow-Up Visit 6[c]
Informed consent	X					
Demographics	X					
Medical history	X					
Current medications and interim medical procedures	X	X		X	X	X
Vital Signs	X	X[h]				
Height	X			X[d]	X[d]	X[d]
Weight	X			X[d]	X[d]	X[d]
Urine, drug & pregnancy screen[a]	X	X				
Breathalyzer Test	X	X				
Childhood Trauma Questionnaire	X					
Randomization	X					
Stop Signal and Tone Tasks		X				
Administer assigned intervention: capsule stimulation		X				
State Trait Anxiety Inventory pre/post intervention	X[b]	X[i]		X[b]	X[b]	X[b]
PANAS-X pre/post intervention	X	X		X	X	X
Interoceptive ratings	X	X				
Anxiety Sensitivity Index-3 pre/post intervention	X	X		X	X	X
PHQ-9	X			X	X	X
IDAS-II Questionnaire	X			X	X	X
NIH PROMIS Anxiety Scales	X			X	X	X
Sheehan Disability Scale	X			X	X	X
Yale-Brown EDS Self-Report (ED YBOCS SRQ)	X					
Big Five Inventory (BFI)	X					
Reward Punishment Sensitivity	X			X	X	X
MAIA Questionnaire	X			X	X	X
Body Scales (Somatomap)	X	X	X			
Body Appreciation Scale 2	X			X	X	X
Body Image States Scale	X			X	X	X
Body Shape Questionnaire	X			X	X	X
EDE-Q Questionnaire	X			X	X	X
Exercise Addiction Inventory	X			X	X	X
Appearance Anxiety Inventory	X			X	X	X
Dysmorphic Concern Questionnaire	X			X	X	X
EDI-3	X			X	X	X
Stunkard Figure Rating Scale	X			X	X	X
Capsule rating scale pre-post intervention		X				
User experience rating scale		X				
Photographic Figure Rating Scale pre/post intervention	X	X		X	X	X
PAGI-SYM Questionnaire	X		X	X	X	X
Meal Emotion Ratings			X			
WHODAS 2.0[e]	X					
GAD-7[e]	X					
DSM-5 Crosscutting assessment (adult)[e]	X					
DSM-5 Crosscutting assessment (youth self report)[f]	X					
DSM-5 Crosscutting assessment (parent/guardian report)[g]	X					
RCADS-25 (youth self report)[f]	X					
RCADS-25 (parent/caregiver report)[g]	X					
Adverse event monitoring	X--X					

[a]Women of childbearing potential [b]State and trait version [c]Assessed via remote data collection, +/- 14 days [d]May be self-reported

[e]Participants 18 and older [f]Participants younger than 18 [g]Parents of participants younger than 18 [h] Collected by inpatient staff [i]STAI state version only

G. Human Participants

We aim to recruit a total of 230 human subjects:

- 115 females with anorexia nervosa (AN), age 15 – 40 years.
- 115 healthy comparisons (HC), females, ages 15 – 40 years, who will be recruited to match to the AN sample based on age.

AN Inclusion criteria:

i. Primary clinical diagnosis of anorexia nervosa as defined by Laureate Eating Disorders Program
ii. Body mass index ≥ 18.5. BMI cannot be below 18.5 within 1 week of capsule swallow.
iii. Transitioned from acute clinical status rating to residential clinical status or partial/intensive outpatient clinical status rating
iv. May be on SSRI and SNRI medications, as well as atypical antipsychotics, tricyclic antidepressants, buspirone, and lamotrigine.
v. No new medication prescription in the week prior to study randomization, Must be on a stable dose of medication for at least 1 week.
vi. Females, ages 15 to 40 years
vii. Women of childbearing age: a hormonal (i.e., oral, implantable, or injectable) and single-barrier method, or a double-barrier method of birth control must be used throughout the study.
viii. Independently ambulatory
ix. Possession of a smartphone with data plan
x. English proficiency
xi. Willingness and ability to participate in study procedures
xii. Provision of signed and dated informed consent form

AN Exclusion criteria:

i. Active suicidal ideation with intent or plan
ii. Active cutting or skin lacerating behaviors
iii. Active purging behaviors (specifically, self-induced vomiting), and/or a history of severe self-induced vomiting
iv. Pregnancy as defined by a urine screen during screening, and confirmed during each stimulation visit, and must not be lactating
v. History of significant gastrointestinal disorder, including any form of inflammatory bowel disease or gastrointestinal malignancy (celiac disease is accepted if the subject has been treated and is in remission)
vi. History of complicated/obstructive diverticular disease
vii. Clinical evidence of significant gastroparesis
viii. Diagnosis of mega-rectum or colon, congenital anorectal malformation, or clinically significant rectocele or rectal prolapse
ix. History of intestinal or colonic obstruction, or suspected intestinal obstruction
x. History of intestinal resection (with an exception for appendectomy, cholecystectomy and inguinal hernia repair), history of bariatric surgery or evidence of any structural abnormality of the gastrointestinal tract that might affect transit

xi. History of Zenker's diverticulum, dysphagia, Barrett's esophagus, esophageal stricture or achalasia, transesophageal fistula, or eosinophilic esophagitis.
xii. Clinical evidence (as judged by the investigator) of respiratory, cardiovascular, renal, hepatic, biliary, endocrine, or neurologic disease
xiii. Chronic use of non-steroidal anti-inflammatory drugs (NSAIDs): chronic use is defined as taking full dose NSAIDs more than three times a week for at least six months. Subjects on cardiac doses of aspirin may be enrolled in the study
xiv. Cardiac pacemaker, implantable cardioverter defibrillator, implantable infusion device, or gastric electrical stimulator
xv. Orthostatic hypotension (defined as a drop of ≥ 20 mm Hg in systolic BP or a drop of ≥ 10 mm Hg in diastolic BP when measured shortly after transitioning from lying down to standing)
xvi. Any other condition which in the opinion of the investigator may adversely affect the safety of the subject or would limit the subject's ability to complete the study
xvii. No smartphone/computer or limited access to a smartphone/computer
xviii. Regular use of any of the following medications or procedures: Medications that may substantially affect intestinal motility, prokinetics at high doses (metoclopramide, erythromycin, senna, prucalopride), anti-Parkinsonian medications, opiates, opioids, calcium-channel blockers, enemas
xix. History of GI bleed within the last 3 months
xx. Pelvic floor dysfunction/defecatory disorder, based on subject history
xxi. Planning to undergo MRI during study time frame
xxii. Any known allergy to soybean or beeswax, or Calcium Carbonate
xxiii. Bradycardia less than 40 beats per minute
xxiv. Pain Disorder

HC Inclusion criteria:

i. Body mass index ≥ 18.5. BMI cannot be below 18.5 within 1 week of capsule swallow.
ii. Females, ages 15 to 40 years
iii. Women of childbearing age: a hormonal (i.e., oral, implantable, or injectable) and single-barrier method, or a double-barrier method of birth control must be used throughout the study.
iv. Independently ambulatory
v. Possession of a smartphone with data plan
vi. English proficiency
vii. Willingness and ability to participate in study procedures
viii. Provision of signed and dated informed consent form

HC Exclusion criteria:

i. Current diagnosis of a psychiatric disorder per the MINI International Diagnostic Interview
ii. Taking any psychotropic medication
iii. Active suicidal ideation with intent or plan
iv. Active cutting or skin lacerating behaviors
v. Active purging behaviors (specifically, self-induced vomiting), and/or a history of severe self-induced vomiting
vi. Pregnancy as defined by a urine screen during screening, and confirmed during each stimulation visit, and must not be lactating

vii. History of significant gastrointestinal disorder, including any form of inflammatory bowel disease or gastrointestinal malignancy (celiac disease is accepted if the subject has been treated and is in remission)
viii. History of complicated/obstructive diverticular disease
ix. Clinical evidence of significant gastroparesis
x. Diagnosis of mega-rectum or colon, congenital anorectal malformation, or clinically significant rectocele or rectal prolapse
xi. History of intestinal or colonic obstruction, or suspected intestinal obstruction
xii. History of intestinal resection (with an exception for appendectomy, cholecystectomy and inguinal hernia repair), history of bariatric surgery or evidence of any structural abnormality of the gastrointestinal tract that might affect transit
xiii. History of Zenker's diverticulum, dysphagia, Barrett's esophagus, esophageal stricture or achalasia, transesophageal fistula, or eosinophilic esophagitis.
xiv. Clinical evidence (as judged by the investigator) of respiratory, cardiovascular, renal, hepatic, biliary, endocrine, or neurologic disease
xv. Chronic use of non-steroidal anti-inflammatory drugs (NSAIDs): chronic use is defined as taking full dose NSAIDs more than three times a week for at least six months. Subjects on cardiac doses of aspirin may be enrolled in the study
xvi. Cardiac pacemaker, implantable cardioverter defibrillator, implantable infusion device, or gastric electrical stimulator
xxv. Orthostatic hypotension (defined as a drop of ≥ 20 mm Hg in systolic BP or a drop of ≥ 10 mm Hg in diastolic BP when measured shortly after transitioning from lying down to standing)
xxvi. Any other condition which in the opinion of the investigator may adversely affect the safety of the subject or would limit the subject's ability to complete the study
xxvii. No smartphone/computer or limited access to a smartphone/computer
xxviii. Regular use of any of the following medications or procedures: Medications that may substantially affect intestinal motility, prokinetics at high doses (metoclopramide, erythromycin, senna, prucalopride), anti-Parkinsonian medications, opiates, opioids, calcium-channel blockers, enemas
xxix. History of a GI bleed within the last 3 months
xxx. Pelvic floor dysfunction/defecatory disorder, based on subject history
xxxi. Planning to undergo MRI during study time frame
xxxii. Any known allergy to soybean or beeswax or Calcium Carbonate
xxxiii. Bradycardia less than 40 beats per minute
xxxiv. Pain Disorder

Plans for recruitment and consent procedures to be followed:

Recruitment

We aim to recruit up to 230 human participants over 5 years (see 'Human Participants' section for details). AN participants will be recruited through clinician referrals from the Laureate Eating Disorders Program. Healthy comparison participants will be recruited through pre-approved LIBR Screening protocols (WIRB # 20250969, WIRB # 20200161, formerly 20101611), through the LIBR database, through radio and internet advertisements, and through word of mouth. Participants who meet our inclusion/exclusion criteria will be offered the opportunity to participate.

Consenting will be conducted in private exam rooms at the Laureate Psychiatric Clinic and Hospital or at LIBR. Consent will be collected by members of the research team that have received training on the consenting process for this study. Each participant will be provided with a full verbal and written explanation of the study purpose, procedures, and risks and benefits. Family members will be allowed to be present and discuss the consenting process with the participant if requested. Sufficient opportunity to review this information and ask questions concerning any aspect of the study will be given.

Compensation for time spent participating

Compensation for participants will be included in the Informed Consent. Participants will be paid with a ClinCard (a pre-paid MasterCard designed for clinical research payments). The ClinCard will be given to the participant at the end of each study visit and funds are available within about 24hrs. Participants will be compensated upon completion of each major study procedure as follows: $50 upon completion of screening enrollment visit, $75 upon completion of Empty Stomach study visit, $75 upon completion of gastric mapping study visit, $150 upon completion of KUB X-ray visit, $25 upon completion of 1 month follow-up, $25 upon completion of 3 month follow-up, and $25 upon completion of 6 month follow-up, for a total compensation of $425 if completing all testing sessions.

H. Risks

Risks associated with self-report questionnaires:

The risks associated with completing the self-report questionnaires are minimal. Some of the questions may be uncomfortable to answer and long questionnaires may elicit boredom. In order to minimize risks associated with the self-report measures, participants will be informed that they may decline to answer any specific questions, or discontinue the interview at any time, that their participation is strictly voluntary and they have the right to withdraw at any time without penalty. The act of measuring body image disturbance can induce distress. We will ensure that any study member assisting in the data collection is a clinician or clinician-in-training approved by Laureate Eating Disorders Program or is supervised by a clinician approved by Laureate Eating Disorders Program.

Risks associated with behavioral tasks:

The risks associated with the stop signal and tone tasks are minimal. Because they require effort some participants may experience mild frustration.

Risks associated with the physiological measures:

All of the physiological measurement devices are commercially available, safe, and non-invasive, posing minimal risk to the participant. Electrodes from the electrocardiogram/electrogastrogram recording may leave a small indent on the participant's body that will dissipate once the device is removed. The electrode gel might leave a sticky residue that can be easily washed off with warm water.

Risks associated with the vibrating capsule:

The following are possible side effects related to the vibrating capsule:

- Abdominal pain/discomfort/cramping
- Blood in the stool may develop or increase
- Bloating/Flatulence
- Diarrhea
- Nausea may develop or increase
- Rectal pain may develop or increase
- Sensation of vibration in the abdomen
- Uncontrolled leakage of stool may occur
- Vomiting may develop
- Bowel obstruction which may result in endoscopic or surgical procedure intervention

Risks associated with the psychophysiological measures:

Measurements of autonomic nervous system activity will be made throughout the experiment. These measurements are made with electrodes placed on the surface of the skin, which can be expected to cause about the same discomfort as might be experienced with wearing an adhesive bandage. The vibrating capsule approach to measuring gastrointestinal perception may induce states of acute anxiety and/or frustration that quickly resolve as soon as the challenge is over. The task might induce some frustration for participants who are unable to feel their gastrointestinal sensations.

Risks associated with the gastric mapping:

Participants may feel uncomfortable due to the Alimetry device's large surface of adhesion, or bored during the 3 hour measurement time period.

Risks associated with the abdominal X-ray:

The amount of radiation exposure associated with each abdominal (KUB) X-ray is quite low (e.g. 0.1 mSV, which is the equivalent of 10 days of natural background radiation). For comparison, other imaging methods such as fluoroscopy involve exposure to much higher amounts of radiation than abdominal X-rays (e.g. 6 mSV which is the equivalent of 2 years of natural background radiation). Thus, participants completing this portion of the experiment will receive 10 KUB X-rays or a total of 1.0 mSV additional radiation exposure (the equivalent of 100 days of natural background radiation). To further reduce the risk of radiation exposure all female participants will undergo urine pregnancy screening and will be excluded from that portion of the study if they are pregnant.

Minimizing risks associated with self report questionnaires:

In order to minimize risks associated with the self-report measures, participants will be informed that they may decline to answer any specific questions, or discontinue the

interview at any time, that their participation is strictly voluntary and they have the right to withdraw at any time without penalty.

Minimizing risks associated with behavioral tasks:

In order to minimize risks associated with the stop signal and tone tasks, participants will be informed that they may discontinue the task at any time, that their participation is strictly voluntary and they have the right to withdraw at any time without penalty.

Minimizing risks associated with psychophysiological testing:

Participants are free to stop the testing at any time, which may immediately remove any feelings of distress.

Minimizing risks associated with the vibrating capsule:

Participants are free to stop the testing at any time, which may immediately remove any feelings of distress. Due to the one-shot programming of the vibrating capsule in the base unit, participants stopping the experiment part of the way through the stimulation will continue to receive the remainder of the stimulations. However, the duration of stimulation is short (<30 minutes in total). Once the stimulation protocol has been completed, the capsule remains inert for the remainder of transit through the gastrointestinal system. The Vibrant capsule has been designated by the US Food and Drug Administration as a 501K De-Novo regulatory path (i.e., non-significant risk device), therefore there is no Investigational Device Exemption filing requirement. This device has been previously approved for research investigation (see WIRB® Protocol #20190204).

Minimizing risks associated with the gastric mapping:

Participants are free to stop the testing at any time, which may immediately remove any feelings of distress.

Potential benefits and importance to the participants:

Participants may not experience any direct benefits from participation. The results of these studies will further the scientific community's understanding of the potential role of gastrointestinal perception and its relationship to symptoms of AN, which may ultimately lead to better tests of illness severity, treatment response prediction, or treatments for eating disorders.

Risk/benefit analysis:

Given that the risks are expected to be minimal with the research but there are no direct benefits to the subjects, the risk/benefit ratio is difficult to calculate. However, the risk/benefit ratio is favorable when considering the results of the research may benefit the ability of future studies to provide better clinical measurements and treatments for individuals with eating disorders.

Plan of Action for Incidental Findings:

Detection and disclosure of incidental findings will be documented in a database contained on the Laureate Institute for Brain Research computer network. Any incidental findings will be shared with the subject and after the subject's approval with their physician.

I. Data Safety Monitoring Plan

A Data Safety Monitoring Plan has been established based on the level of risk and study type. Adverse events and deviations will be made known to the Laureate Institute for Brain Research Human Protection Administrator at (918) 502-5155 or via email at hpa@laureateinstitute.org. All reportable adverse events and/or deviations will be disclosed to the Western IRB.

J. Data Archives

Deidentified study data may be deposited in databases such as the NIMH Data Archive (NDA) where it will be shared with other researchers from around the world. Prospective participants will be allowed to opt out of this data sharing during the consenting process and may also contact study staff at a later date to opt out. However, once data are part of the NDA or another database, the study data that has already been shared cannot be removed.

K. Confidentiality

To protect participant confidentiality, participant data will be anonymized as follows:

All study records that identify the participant (including contact information such as phone number and email address) will be assigned code numbers and will not be individually identifiable. Code numbers are a combination of numbers and letters.

Records of the participant's participation in this study will be held confidential, except when disclosure is required by law, or as described in the informed consent document (under "Confidentiality"). The study doctor, the sponsor or persons working on behalf of the sponsor, and under certain circumstances, the United States Food and Drug Administration (FDA) and the Institutional Review Board (IRB) will be able to inspect and copy confidential study-related records that identify the subject by name. Therefore, absolute confidentiality cannot be guaranteed. If the results of this study are published or presented at meetings, the subject will not be identified.

All research documents containing Personally Identifiable Information (PII) and collected research data will be stored electronically on the LIBR Network and/or REDCap. Access to the LIBR Network and to REDCap is granted only to authorized personnel. The LIBR Network is protected by the Palo Alto PA-5250 Layer 7 firewall with licenses for Wildfire, AV, Threat Prevention, and URL filtering. All LIBR Network data is stored on site. REDCap is a secure web application for building and managing online surveys and

databases. While REDCap can be used to collect virtually any type of data (including 21 CFR Part 11, FISMA, and HIPAA-compliant environments), it is specifically geared to support online or offline data capture for research studies and operations. The REDCap Consortium, a vast support network of collaborators, is composed of thousands of active institutional partners in over one hundred countries who utilize and support REDCap in various ways.

There is risk of possible loss of participant confidentiality. To minimize risk, all study records that identify the participant (including contact information such as phone number and email address) will be assigned code numbers and will not be individually identifiable. Code numbers are a combination of numbers and letters.

The protocol, informed consent document, and relevant supporting information will be submitted to the IRB for review and must be approved before the study is initiated. In addition, any participant recruitment materials must be approved by the IRB prior to use. This study will be conducted in accordance with the ethical principles that have their origin in the Declaration of Helsinki and that are consistent with Good Clinical Practice and applicable regulatory requirements. The study must be conducted in accordance with the regulations of the United States Food and Drug Administration (FDA) as described in 21 CFR 50 and 56, applicable laws and the IRB requirements.

The sponsor will submit any change to the protocol to the IRB for review and approval before implementation. A protocol change intended to eliminate an apparent immediate hazard to participants may be implemented immediately, provided the reviewing IRB is notified within 5 working days.

It is the responsibility of the investigator to provide each subject with full and adequate verbal and written information, before inclusion in the study, using the IRB approved informed consent document, including the objective and procedures of the study and the possible risks involved. Informed consent must be obtained prior to performing any study-related procedures. A copy of the signed informed consent document will be given to the study subject.

References:

Arcelus J, Mitchell AJ, Wales J, Nielsen S (2011) Mortality rates in patients with anorexia nervosa and other eating disorders. A meta-analysis of 36 studies. Archives of general psychiatry 68:724-731.

Aziz Q, Andersson JL, Valind S, Sundin A, Hamdy S, Jones AK, Foster ER, Langstrom B, Thompson DG (1997) Identification of human brain loci processing esophageal sensation using positron emission tomography. Gastroenterology 113:50-59.

Aziz Q, Schnitzler A, Enck P (2000a) Functional neuroimaging of visceral sensation. J Clin Neurophysiol 17:604-612.

Aziz Q, Thompson DG, Ng VWK, Hamdy S, Sarkar S, Brammer MJ, Bullmore ET, Hobson A, Tracey I, Gregory L, Simmons A, Williams SCR (2000b) Cortical processing of human somatic and visceral sensation. The Journal of Neuroscience 20:2657-2663.

Benini L, Todesco T, Dalle Grave R, Deiorio F, Salandini L, Vantini I (2004) Gastric emptying in patients with restricting and binge/purging subtypes of anorexia nervosa. Am J Gastroenterol 99:1448-1454.

Berner LA, Simmons AN, Wierenga CE, Bischoff-Grethe A, Paulus MP, Bailer UF, Ely AV, Kaye WH (2017) Altered interoceptive activation before, during, and after aversive breathing load in women remitted from anorexia nervosa. Psychol Med 1-13.

Bluemel S, Menne D, Milos G, Goetze O, Fried M, Schwizer W, Fox M, Steingoetter A (2017) Relationship of body weight with gastrointestinal motor and sensory function: studies in anorexia nervosa and obesity. BMC Gastroenterol 17:4.

Bohon C, Stice E, Spoor S (2009) Female emotional eaters show abnormalities in consummatory and anticipatory food reward: a functional magnetic resonance imaging study. The International journal of eating disorders 42:210-221.

Coddington RD, Bruch H (1970) Gastric perceptivity in normal, obese and schizophrenic subjects. Psychosomatics: Journal of Consultation and Liaison Psychiatry 11:571-579.

Diamanti A, Bracci F, Gambarara M, Ciofetta GC, Sabbi T, Ponticelli A, Montecchi F, Marinucci S, Bianco G, Castro M (2003) Gastric electric activity assessed by electrogastrography and gastric emptying scintigraphy in adolescents with eating disorders. J Pediatr Gastroenterol Nutr 37:35-41.

Frank GK, Oberndorfer TA, Simmons AN, Paulus MP, Fudge JL, Yang TT, Kaye WH (2008) Sucrose activates human taste pathways differently from artificial sweetener. Neuroimage 39:1559-1569.

Gharibans AA, Kim S, Kunkel D, Coleman TP (2017) High-Resolution Electrogastrogram: A Novel, Noninvasive Method for Determining Gastric Slow-Wave Direction and Speed. IEEE Trans Biomed Eng 64:807-815.

Gharibans AA, Smarr BL, Kunkel DC, Kriegsfeld LJ, Mousa HM, Coleman TP (2018) Artifact Rejection Methodology Enables Continuous, Noninvasive Measurement of Gastric Myoelectric Activity in Ambulatory Subjects. Sci Rep 8:5019.

Halmi KA, Sunday SR (1991) Temporal patterns of hunger and fullness ratings and related cognitions in anorexia and bulimia. Appetite 16:219-237.

Hassanpour MS, Simmons WK, Feinstein JS, Luo Q, Lapidus RC, Bodurka J, Paulus MP, Khalsa SS (2018) The insular cortex dynamically maps changes in cardiorespiratory interoception. Neuropsychopharmacology 43:426-434.

Herbert BM, Muth ER, Pollatos O, Herbert C (2012) Interoception across modalities: On the relationship between cardiac awareness and the sensitivity for gastric functions. PLoS ONE 7.

Heruc GA, Little TJ, Kohn M, Madden S, Clarke S, Horowitz M, Feinle-Bisset C (2018) Appetite Perceptions, Gastrointestinal Symptoms, Ghrelin, Peptide YY and State Anxiety Are Disturbed in Adolescent Females with Anorexia Nervosa and Only Partially Restored with Short-Term Refeeding. Nutrients 11.

Hobday DI, Hobson A, Furlong PL, Thompson DG, Aziz Q (2000) Comparison of cortical potentials evoked by mechanical and electrical stimulation of the rectum. Neurogastroenterol Motil 12:547-554.

Hobson AR, Sarkar S, Furlong PL, Thompson DG, Aziz Q (2000) A cortical evoked potential study of afferents mediating human esophageal sensation. Am J Physiol Gastrointest Liver Physiol 279:G139-147.

Holzl R, Erasmus LP, Moltner A (1996) Detection, discrimination and sensation of visceral stimuli. Biol Psychol 42:199-214.

Kerr KL, Moseman SE, Avery JA, Bodurka J, Zucker NL, Simmons WK (2016) Altered Insula Activity during Visceral Interoception in Weight-Restored Patients with Anorexia Nervosa. Neuropsychopharmacology 41:521-528.

Khalsa SS, Adolphs R, Cameron OG, Critchley HD, Davenport PW, Feinstein JS, Feusner JD, Garfinkel SN, Lane RD, Mehling WE, Meuret AE, Nemeroff CB, Oppenheimer S, Petzschner FH, Pollatos O, Rhudy JL, Schramm LP, Simmons WK, Stein MB, Stephan KE, Van den Bergh O, Van Diest I, von Leupoldt A, Paulus MP, Interoception Summit p (2018a) Interoception and Mental Health: A Roadmap. Biological psychiatry : cognitive neuroscience and neuroimaging 3:501-513.

Khalsa SS, Craske MG, Li W, Vangala S, Strober M, Feusner JD (2015) Altered interoceptive awareness in anorexia nervosa: Effects of meal anticipation, consumption and bodily arousal. The International journal of eating disorders 48:889-897.

Khalsa SS, Hassanpour MS, Strober M, Craske MG, Arevian AC, Feusner JD (2018b) Interoceptive Anxiety and Body Representation in Anorexia Nervosa. Frontiers in psychiatry 9:444.

Naliboff BD, Berman S, Suyenobu B, Labus JS, Chang L, Stains J, Mandelkern MA, Mayer EA (2006) Longitudinal change in perceptual and brain activation response to visceral stimuli in irritable bowel syndrome patients. Gastroenterology 131:352-365.

Peterson CM, Tissot AM, Matthews A, Hillman JB, Peugh JL, Rawers E, Tong J, Mitan L (2016) Impact of short-term refeeding on appetite and meal experiences in new onset adolescent eating disorders. Appetite 105:298-305.

Robinson PH (1989) Perceptivity and paraceptivity during measurement of gastric emptying in anorexia and bulimia nervosa. Br J Psychiatry 154:400-405.

Ron Y, Halpern Z, Safadi R, Dickman R, Dekel R, Sperber AD (2015) Safety and efficacy of the vibrating capsule, an innovative non-pharmacological treatment modality for chronic constipation. Neurogastroenterol Motil 27:99-104.

Salet GA, Samsom M, Roelofs JM, van Berge Henegouwen GP, Smout AJ, Akkermans LM (1998) Stomach distension in extremely obese and in normal subjects. Gut 42:823-829.

Salvioli B, Pellicciari A, Iero L, Di Pietro E, Moscano F, Gualandi S, Stanghellini V, De Giorgio R, Ruggeri E, Franzoni E (2013) Audit of digestive complaints and psychopathological traits in patients with eating disorders: a prospective study. Dig Liver Dis 45:639-644.

Sato Y, Fukudo S (2015) Gastrointestinal symptoms and disorders in patients with eating disorders. Clin J Gastroenterol 8:255-263.

Sheehan DV, Lecrubier Y, Sheehan KH, Amorim P, Janavs J, Weiller E, Hergueta T, Baker R, Dunbar GC (1998) The Mini-International Neuropsychiatric Interview (M.I.N.I.): the development and validation of a structured diagnostic psychiatric interview for DSM-IV and ICD-10. The Journal of clinical psychiatry 59 Suppl 20:22-33;quiz 34-57.

Stice E, Spoor S, Bohon C, Small DM (2008) Relation between obesity and blunted striatal response to food is moderated by TaqIA A1 allele. Science 322:449-452.

Wagner A, Aizenstein H, Mazurkewicz L, Fudge J, Frank GK, Putnam K, Bailer UF, Fischer L, Kaye WH (2008) Altered insula response to taste stimuli in individuals recovered from restricting-type anorexia nervosa. Neuropsychopharmacology 33:513-523.
