# Supplementary material for: Gastrointestinal Interoception and Relapse in Anorexia Nervosa
Source: JAMA Psychiatry. 2026 Jun 17:e261301. Online ahead of print. doi: 10.1001/jamapsychiatry.2026.1301 (PMC13276667; doi:10.1001/jamapsychiatry.2026.1301)
Supplement: Supplement 2. — eMethods 1. Participants eTable 1. Diagnostic comorbidities, psychotropic medication status, and race of anorexia nervosa (AN) and healthy comparison (HC) participants eTable 2. Exclusion criteria eMethods 2. Experimental protocol eFigure 1. Experimental setup eMethods 3. Behavioral and self-report measures eMethods 4. Computational modeling eTable 3. Prior means for key model parameters eTable 4. Overview of model variants and their corresponding parameters eMethods 5. Processing of peripheral physiological data eMethods 6. Illness status definitions eMethods 7. Statistical analysis eResults 1. Behavioral and self-report findings eFigure 2. Retrospective ratings of interoceptive sensations and homeostatic urges before, during, and after vibratory gut stimulation eResults 2. Electroencephalogram findings eFigure 3. Group overlay of onset-evoked gastric-evoked potential (GEP) responses eFigure 4. Group overlay of offset-evoked gastric-evoked potential (GEP) responses eResults 3. Computational findings eTable 5. Model comparison results eTable 6. Correlation results for parameter recoverability analysis eFigure 5. Prior time-course eResults 4. Longitudinal findings eFigure 6. Individual trajectories of eating disorder symptoms from discharge to 6-month follow-up in AN eTable 7. Logistic regression results predicting 6-month AN status (full relapse) from experimental session measures eTable 8. Self-report measures: logistic regression predicting 6-month AN illness status (full relapse) from retrospective ratings of sensation intensity and valence (unpleasant/pleasant) and state items before, during, and after vibratory gut stimulation eTable 9. Linear regression results predicting EDE-Q Total score at 6 months from experimental session measures eTable 10. Self-report measures: linear regression results predicting EDE-Q Total score at 6 months from retrospective ratings of sensation intensity and valence (unpleasant/pleasant) and state items before, during, and after vi [file jamapsychiatry-e261301-s002.pdf]

Supplemental Online Content

Verdonk C, Mink K, Choquette E, et al. Gastrointestinal interoception and relapse in anorexia nervosa. *JAMA Psychiatry*. Published online June 17, 2026.
doi:10.1001/jamapsychiatry.2026.1301

eMethods 1. Participants
eTable 1. Diagnostic comorbidities, psychotropic medication status, and race of anorexia nervosa (AN) and healthy comparison (HC) participants
eTable 2. Exclusion criteria
eMethods 2. Experimental protocol
eFigure 1. Experimental setup
eMethods 3. Behavioral and self-report measures
eMethods 4. Computational modeling
eTable 3. Prior means for key model parameters
eTable 4. Overview of model variants and their corresponding parameters
eMethods 5. Processing of peripheral physiological data
eMethods 6. Illness status definitions
eMethods 7. Statistical analysis
eResults 1. Behavioral and self-report findings
eFigure 2. Retrospective ratings of interoceptive sensations and homeostatic urges before, during, and after vibratory gut stimulation
eResults 2. Electroencephalogram findings
eFigure 3. Group overlay of onset-evoked gastric-evoked potential (GEP) responses
eFigure 4. Group overlay of offset-evoked gastric-evoked potential (GEP) responses
eResults 3. Computational findings
eTable 5. Model comparison results
eTable 6. Correlation results for parameter recoverability analysis
eFigure 5. Prior time-course
eResults 4. Longitudinal findings
eFigure 6. Individual trajectories of eating disorder symptoms from discharge to 6-month follow-up in AN
eTable 7. Logistic regression results predicting 6-month AN status (full relapse) from experimental session measures
eTable 8. Self-report measures: logistic regression predicting 6-month AN illness status (full relapse) from retrospective ratings of sensation intensity and valence (unpleasant/pleasant) and state items before, during, and after vibratory gut stimulation
eTable 9. Linear regression results predicting EDE-Q Total score at 6 months from experimental session measures
eTable 10. Self-report measures: linear regression results predicting EDE-Q Total score at 6 months from retrospective ratings of sensation intensity and valence (unpleasant/pleasant) and state items before, during, and after vibratory gut stimulation
eResults 5. Peripheral physiological findings
eFigure 7. Peripheral physiological responses during baseline and vibratory gut stimulation
eResults 6. Multilevel correlation findings

© 2026 Verdonk C et al. *JAMA Psychiatry*.

eTable 11. Correlations between average gastric-evoked potential (avGEP) amplitude and behavioral or computational measures for the normal and enhanced stimulation blocks
eDiscussion
eReferences

This supplemental material has been provided by the authors to give readers additional information about their work.

© 2026 Verdonk C et al. *JAMA Psychiatry*.

eMethods 1. Participants

1. Participant flow

One hundred eighty-seven participants provided informed written consent and were assessed for eligibility. Thirty-two participants were excluded due to exclusionary medical conditions (see Section 1: Exclusion Criteria below), and an additional nine participants withdrew prior to the intervention for personal reasons. Of the 146 participants allocated to the intervention, 78 individuals were diagnosed with anorexia nervosa (AN) restrictive subtype and 68 individuals were defined as healthy comparisons (HCs). One AN participant and one HC participant withdrew before any data collection due an inability to swallow the capsule. After completion of the intervention, data related to 25 participants were discarded for the following reasons: inadequate behavioral response registration (n=4), corrupted behavioral data file (n=1), corrupted electroencephalography (EEG) data files (n=5), low quality gastric-evoked potential (GEP) signal (n=9), and low-quality capsule signal (n=6). For follow-up data, 6-month results from 8 individuals with AN were missing due to passive refusals (not responding to remote follow-up requests) (Figure 1). The data were collected from August 15, 2020 to December 05, 2024 at the Laureate Institute for Brain research (Tulsa, Oklahoma).

2. Exclusion criteria

Participants were excluded from the study if they had a diagnosis of a psychosis-spectrum disorder or bipolar disorder, or endorsed severe behavioral disturbances such as active suicidal ideation, recent self-harm behavior, active purging, or a severe history of purging. Pregnancy or lactation was also exclusionary. Gastrointestinal-related exclusions included diagnoses such as inflammatory bowel disease, gastroparesis, complicated diverticular disease, intestinal obstruction, mega-rectum or colon, congenital anorectal malformation, and rectal prolapse (Table S2). Participants with a history of intestinal resection (excluding appendectomy, cholecystectomy, or inguinal hernia repair), bariatric surgery, or other structural abnormalities that could impact gastrointestinal transit were excluded. Additionally, individuals with esophageal disorders (such as Zenker's diverticulum, dysphagia, Barrett's esophagus, achalasia, or eosinophilic esophagitis) were not eligible. Cardiovascular exclusion criteria included orthostatic hypotension and bradycardia defined as a heart rate below 40 beats per minute. Pain disorders were also exclusionary. Finally, chronic use of non-steroidal anti-inflammatory drugs, defined as taking full dose NSAIDs more than three times a week for at least six months, and regular use of medications known to significantly alter gastrointestinal motility—such as high-dose prokinetics (e.g., metoclopramide, erythromycin, senna, prucalopride), anti-Parkinsonian agents, opioids, calcium-channel blockers, or frequent use of enemas—were grounds for exclusion.

Participants with a history of severe purging were excluded out of an abundance of caution, given the novel use of an ingestible vibrating capsule to probe gut mechanosensation in this population. While the capsule is non-invasive and generally well tolerated, we aimed to mitigate any potential risk of gastrointestinal complications. Severe purging behaviors (particularly those involving self-induced vomiting) have been associated with structural weakening of the gastrointestinal tract, including risks of gastroesophageal rupture, mucosal damage, or delayed gastric emptying[1,2]. Even in the absence of current behaviors, a history of severe purging may confer residual physiological vulnerability. Thus, in this initial study, we prioritized participant safety by excluding individuals with significant past purging histories. Future studies, once feasibility and safety are further established, may consider extending inclusion to individuals with more active or severe purging profiles to enhance generalizability.

3. Participant compensation

All participants received financial compensation via a ClinCard (pre-paid debit card), with payments prorated per study visit and issued upon completion of each major procedure. Participants were eligible to receive up to $425 for full completion of all study visits and follow-up assessments.

© 2026 Verdonk C et al. *JAMA Psychiatry*.

eTable 1. Diagnostic comorbidities, psychotropic medication status, and race of anorexia nervosa (AN) and healthy comparison (HC) participants.

	AN (n=62 females)	HC (n=57 females)
**Comorbid Diagnoses** - N (%)		
Generalized anxiety disorder	43 (69)	0
Major depressive disorder	23 (37)	0
Obsessive–compulsive disorder	12 (19)	0
**Psychotropic medication** - N (%)	57 (92)	
Adrenergic antagonist[1]	2 (3)	
Dopamine modulator[2]	19 (31)	
GABA modulator[3]	3 (5)	
Glutamate modulator[4]	5 (8)	
Histamine antagonist[5]	23 (37)	0
Lithium enzyme modulator[6]	1 (2)	
Melatonin agonist[7]	21 (34)	
Norepinephrine modulator[8]	19 (31)	
Opioid antagonist[9]	1 (2)	
Serotonin modulator[10]	57 (92)	
**Race**		
Asian	2 (3)	9 (16)
Black/African American	0	3 (5)
Middle Eastern	1 (2)	0
Native American	3 (5)	1 (2)
Other	0	2 (3)
White	56 (90)	42 (74)

M: mean; SD: standard deviation; N: number of participants; %: proportion of participants. Race was self-reported by participants by selecting from a list of options provided by the investigator team (copied from the National Institutes of Health list of racial categories).

[1]propanolol; [2]amantadine, aripiprazole, brexpiprazole, bupropion, cariprazine, lurasidone, olanzapine, risperidone; [3]lorazepam, melatonin-GABA-valerian supplement; [4]gabapentin, lamotrigine; [5]cetirizine, diphenhydramine, doxepin, fexofenadine, hydroxyzine, levocetirizine, loratadine, quetiapine ; [6]lithium carbonate; [7]melatonin supplement, melatonin-GABA-valerian supplement; [8]atomoxetine, amitriptyline, bupropion, desvenlafaxine, duloxetine, mirtazapine, risperidone, venlafaxine; [9]naltrexone; [10]amitriptyline, aripiprazole, brexpiprazole, buspirone, cariprazine, desvenlafaxine, duloxetine, escitalopram, fluoxetine, fluvoxamine, lurasidone, mirtazapine, olanzapine, risperidone, sertraline, trazodone, venlafaxine, vilazodone.

© 2026 Verdonk C et al. *JAMA Psychiatry*.

eTable 2. Exclusion criteria.

Exclusionary condition
Psychosis spectrum disorder or bipolar disorder
Active suicidal ideation, engagement in self-harming behaviors, ongoing purging behaviors, or a severe history of purging
Pregnancy and lactation
Significant gastrointestinal disorder, including any form of inflammatory bowel disease or gastrointestinal malignancy (celiac disease is accepted if the subject has been treated and is in remission)
Complicated/obstructive diverticular disease
Gastroparesis
Mega-rectum or colon, congenital anorectal malformation, or clinically significant rectocele or rectal prolapse
Intestinal or colonic obstruction, or suspected intestinal obstruction
Intestinal resection (with an exception for appendectomy, cholecystectomy and inguinal hernia repair), bariatric surgery or evidence of any structural abnormality of the gastrointestinal tract that might affect transit
Zenker's diverticulum, dysphagia, Barrett's esophagus, esophageal stricture or achalasia, transesophageal fistula, or eosinophilic esophagitis.
Clinical evidence (as judged by the investigator) of respiratory, cardiovascular, renal, hepatic, biliary, endocrine, or neurologic disease
Orthostatic hypotension
Gastrointestinal bleed within the last 3 months
Pelvic floor dysfunction/defecatory disorder
Bradycardia with resting heart rate less than 40 bpm
Pain disorder
Known allergy to soybeans, beeswax, or calcium carbonate
**Exclusionary medication**
Chronic use of non-steroidal anti-inflammatory drugs (NSAIDs), defined as taking full dose NSAIDs more than three times a week for at least six months. Those taking cardiac (i.e., low) doses of aspirin may be enrolled
Regular use of any of the following medications or procedures: Medications that may substantially affect intestinal motility, prokinetics at high doses (metoclopramide, erythromycin, senna, prucalopride), anti-Parkinsonian medications, opiates, opioids, calcium-channel blockers, enemas

4. Gastrointestinal Symptoms and Diagnoses

Gastrointestinal conditions such as gastroparesis or inflammatory bowel disease (IBD) were exclusion criteria in the present study for safety and tolerability reasons related to the capsule protocol. Specifically, gastroparesis is associated with altered gastric motility, which we thought might confound measures of GI perceptual accuracy, while IBD (which includes conditions such as Crohn's Disease or Ulcerative Colitis)

cause structural inflammatory alterations of the gastrointestinal tract (e.g., skip lesions in the small and large bowel in Crohn's disease vs. distal colon wall weakening in Ulcerative Colitis) that raised potential safety concerns regarding capsule transit or retention. In contrast, irritable bowel syndrome (IBS) was not an exclusion criterion, as it is a functional disorder without structural abnormalities that would pose a risk for capsule passage.

Gastrointestinal (GI) symptoms and diagnoses were systematically assessed at intake in both groups using structured self-report questionnaires. Assessed symptoms included abdominal pain, nausea or vomiting, stomach or intestinal problems, frequent diarrhea, indigestion, and evidence of gastrointestinal bleeding (blood in stool or vomiting blood). Diagnoses assessed included celiac disease, peptic ulcer disease, liver disease, hepatitis, esophageal varices, jaundice, and ulcerative colitis.
In the AN group, GI symptoms were common, including abdominal pain (28/62), nausea or vomiting (17/62), indigestion (14/62), and stomach or intestinal problems (13/62). Less frequent findings included blood in stool (2/62), celiac disease (1/62), and stomach ulcer (1/62). No participants with AN reported frequent diarrhea, vomiting blood, liver disease, jaundice, esophageal varices, or ulcerative colitis.
In healthy controls, GI symptoms were infrequent, with abdominal pain reported in 6/57, nausea or vomiting in 2/57, blood in stool in 2/57, and hepatitis in 2/57. No healthy controls reported indigestion, stomach or intestinal problems, or structural gastrointestinal disorders.
Importantly, IBD and other structural gastrointestinal conditions that could pose safety concerns for capsule transit were not present in either group.".

© 2026 Verdonk C et al. *JAMA Psychiatry*.

eMethods 2. Experimental protocol

1. Meal protocol

Participants were instructed to fast from both food and liquids (excluding water) for at least three hours prior to the capsule session and were informed they would not be permitted to eat or drink during the visit. This fasting protocol was implemented to allow gastric emptying to occur and to reduce baseline gastrointestinal activity prior to capsule-related measurements. For participants with AN, fasting and scheduling were coordinated with their inpatient treatment program. AN participants ate lunch at 12:00 PM on the unit, attended the study session from 2:00 to 5:00 PM (with the capsule swallow task occurring at approximately 4:00 PM), and returned to the inpatient unit following the appointment to eat dinner at 5:00 PM in accordance with their meal plan. Thus, AN participants were in a pre-meal phase during capsule stimulations, with the expectation that they would eat a meal upon completion of the experiment. HC participants followed the same fasting protocol but were scheduled independently.

2. Vibrating capsule

The Vibrant capsule, developed by Vibrant Ltd, has received marketing authorization by the Food and Drug Administration for the treatment of adults with chronic idiopathic constipation by delivering mechanical stimulation directly to the colon. It is an orally administered, non-biodegradable device that can be wirelessly activated using a specialized activation unit (see eFigure 1). The Vibrant capsule is classified as a non-significant risk (NSR) device, and its safety has been validated in studies involving both healthy volunteers[3,4] and patients with chronic constipation.[5,6]

3. Masking procedure

To manage participants' expectations, they were informed that two different versions of the Vibrant capsule were being tested and that they would be randomly assigned to one of three study groups: two involving vibrating capsules (modes A or B) or a non-vibrating placebo capsule. Participants were also told that neither they nor the experimenter would know whether any stimulation would occur. In reality, all participants received a vibrating capsule, making this a single-blinded study. All participants were instructed to fast for three hours before the study to ensure an empty stomach, based on the rationale that a healthy person without GI disorders would likely have an empty stomach three hours after eating.

4. Mechanosensory stimulation

Mechanosensory stimulations began shortly after participants ingested the activated Vibrant capsule, which was swallowed with approximately 240 ml (8 ounces) of water while they sat comfortably in a chair. During the session, they were instructed to focus their attention on abdomen sensations while fixating on a cross on a monitor positioned about 60 cm away. They used their dominant hand to press a button when they detected the capsule's vibrations and released it once the sensation ended. Stimulations commenced approximately three minutes after the capsule's activation. Participants remained seated throughout the experiment to reduce motion artifacts in the EEG and EGG recordings. They were advised to keep their non-dominant hand on their lap and avoid touching their abdomen, while a research assistant seated behind them in the room monitored their alertness and adherence to instructions. Each participant underwent two blocks of stimulation (normal and enhanced) in a counterbalanced order, with each block delivering 60 stimulations over a 13-minute period. A 4-minute break separated the blocks, resulting in a total stimulation period of approximately 33 minutes.

5. Vibration detection

To accurately record the timing of each vibration, a digital stethoscope (Thinklabs Inc.) was affixed to the lower right quadrant of the abdomen using a Tegaderm patch (15 x 20 cm). The stethoscope signal was continuously recorded throughout the experiment at a sampling rate of 1000 Hz. Custom scripts in Matlab 2021a (Mathworks®) were developed to identify the onset and offset of each vibration. The timing of each vibration was then manually verified and adjusted if necessary. Participants whose vibration amplitudes could not be reliably detected due to technical issues were excluded from the analysis (Figure 1).

eFigure 1. Experimental setup.

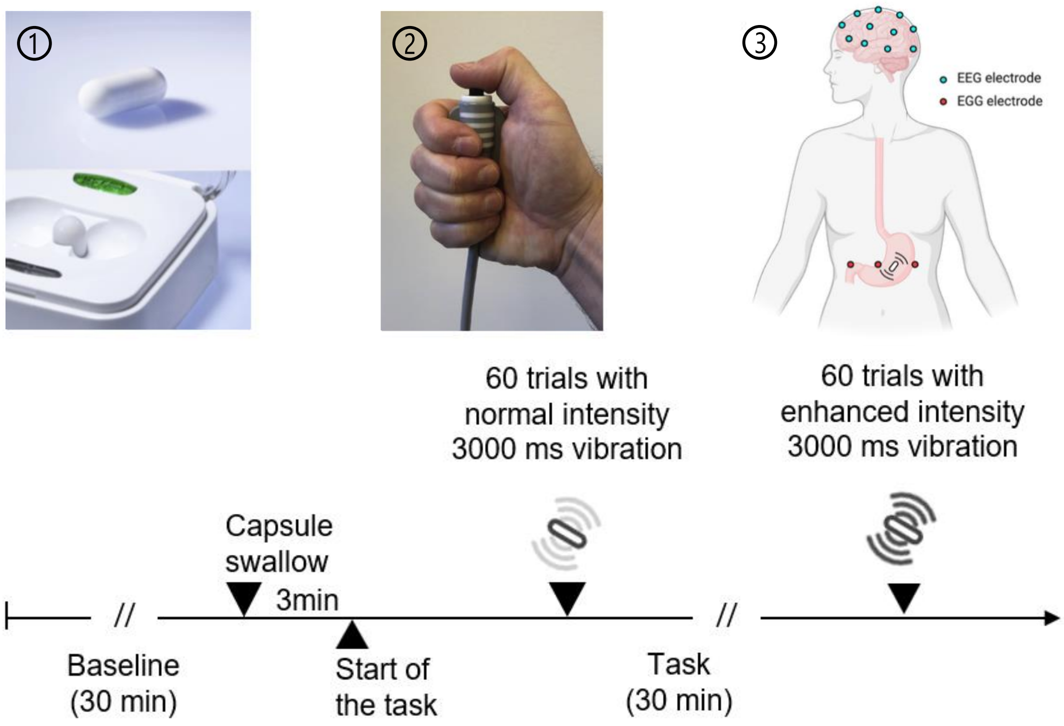

The gastrointestinal (GI) detection task included two counterbalanced blocks of stimulation (normal and enhanced) generated by the vibrating capsule (Vibrant Ltd). The capsule was activated just before swallowing by placing it in the activation base (①). Each block included approximately 60 stimulations, each lasting 3 seconds. Participants were instructed to continuously attend to their stomach/digestive system and to press and hold a button (②) as soon as they felt a sensation attributed to the capsule and to release the button as soon as the sensation ended. During the GI detection task, neural (electroencephalogram), gastric (electrogastrogram), cardiac (electrocardiogram), galvanic (skin conductance), and subjective responses were recorded simultaneously (③). Abbreviations: ms, milliseconds; min, minutes.

© 2026 Verdonk C et al. *JAMA Psychiatry*.

eMethods 3. Behavioral measures and self-report measures

1. Behavioral measures

We calculated perceptual accuracy for each participant and experimental block using *A-prime (A′),* a non-parametric signal detection index suitable for designs with fewer trials.[7,8] Higher A' values indicate better discrimination between signal-present and signal-absent trials. Values were normalized as in our prior work[3] to fall between 0 and π, where π corresponds to maximum accuracy. A′ was computed as shown in Equation 1:

$$\mathrm{A}' = \begin{cases} 0.5 + \dfrac{(H-F)(1+H-F)}{4H(1-F)} & when\ H \geq F \\ 0.5 - \dfrac{(F-H)(1+F-H)}{4F(1-H)} & when\ H < F \end{cases} \tag{1}$$

where H represents the hit rate and F denotes the false-alarm rate.

The A' scores were then normalized using Equation 2, yielding values between 0 and π.

$$\text{Normalized A Prime} = 2\sin^{-1}\left(\sqrt{A'}\right) \tag{2}$$

We also calculated response bias, which captures the overall tendency to press versus not press the button. Positive values (ranging from 0 to 1) indicate a tendency to respond 'no' (i.e., not press the button), whereas negative values (ranging from -1 to 0) indicate a liberal tendency to respond 'yes' (i.e., press the button). A' and response bias were reported separately to distinguish perceptual sensitivity from response strategy, following standard signal detection theory approaches. [7,8] While A' is intended to capture discrimination ability between signal-present and signal-absent trials, it is derived from the same hit and false-alarm rates as response bias measures and therefore should not be interpreted as mathematically independent of response tendency. No response-bias correction was applied to A'; accordingly, response tendency could still influence group differences in A'. A' and response bias were therefore analyzed and reported as complementary indices of interoceptive decision-making. Response bias was computed as follows (Equation 3):

$$\mathrm{B}'' = \begin{cases} \dfrac{H(1-H)-F(1-F)}{H(1-H)+F(1-F)} & when\ H \geq F \\ \dfrac{F(1-F)-H(1-H)}{F(1-F)+H(1-H)} & when\ H < F \end{cases} \tag{3}$$

where H represents the hit rate and F denotes the false-alarm rate.

Additional behavioral measures included the miss rate and response time, the latter defined as the difference between the onset of the vibration and the participant's button press, indicating their perception of the sensation.

2. Self-report measures

Before and after the task, participants completed visual analog scales (0–100). Retrospective items assessed intensity and valence for the stomach/digestive system, breath, and heartbeat; muscle tension was assessed for intensity only. State items assessed current hunger, thirst, and urges to urinate/defecate.

Pre-task stems referenced the past hour (e.g., "Over the past hour, how intensely did you feel your [stomach/digestive system/breath/heartbeat]?"; "…how pleasant or unpleasant were your [same]?"), and post-task stems referenced the stimulation period (e.g., "During the capsule stimulation, how intensely…?"; "…how pleasant or unpleasant…?"). For muscle tension, the pre-task item used a current ("right now") stem and the post-task item used a retrospective ("during capsule stimulation") stem. State items used identical wording at both time points (e.g., "How [hungry/thirsty/much of an urge to urinate/defecate] do you currently feel?").

Scale anchors were: intensity/state 0 = "Not at all/None," 100 = "Extremely/The most I have ever felt," and valence 0 = "Extremely unpleasant," 100 = "Extremely pleasant."

© 2026 Verdonk C et al. *JAMA Psychiatry*.

eMethods 4. Computational modeling

1. Computational model

To evaluate gastrointestinal (GI) interoception and participant's behavior in the experimental task, we used a Bayesian model of perception derived from a Markov decision process (MDP) formulation of active inference.[9-11] Matlab code used to build this model and fit parameters to behavioral data can be accessed at https://github.com/rssmith33/Gut-Inference-Model-Scripts.

Each trial in the model represented a 3-second window during which participants were informed that a vibration could occur. Observations (o) were categorical, consisting of vibration, no-vibration, and a formal trial "start" observation. The hidden states (s), representing the participant's perception, were also categorical and included vibration, no-vibration, and a "start" state (paired with the "start" observation). Every trial was structured with two timesteps ($t = 1$ and $t = 2$). At $t = 1$, participants always began in the "start" state and made a corresponding "start" observation. At $t = 2$, they either received a vibration or no-vibration observation and inferred whether they had transitioned from the "start" state to either the no-vibration or vibration state. Specifically, by combining their prior beliefs about the transition probabilities from the "start" state $p(s_{t=2}|s_{t=1})$(encoded in a matrix **B**) with their beliefs about the precision of the mapping between vibration states and observations, $p(o_{t=2}|s_{t=2})$(encoded in a matrix **A**), they calculated a posterior distribution over states $p(s_{t=2}|o_{t=2})$ as follows:

$$p(s_{t=2}|o_{t=2}) = \boldsymbol{\sigma}(\ln \mathbf{B}\, p(s_{t=1}) + \ln \mathbf{A}^{\mathrm{T}}\, o_{t=2}) \quad (4)$$

Here, σ indicates a softmax function returning the resulting vector back to a proper probability distribution.

The matrix **B** encodes the probability of transitioning from the "start" state to either the "vibration" (pV) or "no vibration" ($1 - pV$) state, as defined in Equation 5. When $pV > 0.5$, it reflects a prior belief that transitions to the vibration state are more probable (e.g., expecting many vibrations throughout the task). Conversely, when $pV < 0.5$, it suggests a prior belief that transitions to the "vibration" state are less likely (e.g., expecting a low vibration frequency throughout the task).

$$\mathbf{B} = p(s_{t=2}|s_{t=1}) = \begin{bmatrix} 0 & 0 & 0 \\ 1 - pV & 1 & 0 \\ pV & 0 & 1 \end{bmatrix} \quad (5)$$

Here, the columns (from left to right) represent the "start" state, "no-vibration" state, and "vibration" state at time $t = 1$, while the rows (from top to bottom) correspond to the same states at time $t = 2$. Additionally, the second and third columns indicate that once an individual reaches either the vibration or no-vibration state, the state remains unchanged within the trial (where a trial is defined by the 3-second time window in which a vibration either was or was not presented).

The matrix **A** encoded the likelihood of receiving a vibration (IP) or no-vibration ($1 - IP$) observation, given the presence of the no-vibration or vibration state (Equation 6). When $IP = 0.5$, this indicates minimal precision, meaning that the probability of detecting a vibration or no-vibration is 0.5, regardless of the actual state. Conversely, an IP value approaching 1 reflects high precision, where the probability of perceiving a vibration is high in the vibration state and low in the no-vibration state (and vice versa for no-vibration).

$$\mathbf{A} = p(o_t|s_t) = \begin{bmatrix} 1 & 0 & 0 \\ 0 & IP & 1 - IP \\ 0 & 1 - IP & IP \end{bmatrix} \quad (6)$$

Similarly, the columns (from left to right) represent the "start" state, "no-vibration" state, and "vibration" state, while the rows (from top to bottom) correspond to the "start" observation, "no-vibration" observation, and "vibration"

observation. The likelihood of perceiving either a vibration or no-vibration given the actual state was determined by the interoceptive precision (IP) parameter. When IP = 0.5, it indicates minimal precision, meaning that the probability of detecting a vibration or no-vibration is 0.5, regardless of the actual state. Conversely, an IP value approaching 1 reflects high precision, where the probability of perceiving a vibration is high in the vibration state and low in the no-vibration state (and vice versa for no-vibration).

At each trial, the participant response involved two possible actions: pressing or not pressing the button. Our model assumed that the probability of choosing to press the button reflected the posterior probability assigned to the vibration state (versus the no vibration state) at time $t = 2$, as defined in Equation 7:

$$P(press) = \mathbf{P}(\boldsymbol{s_{t=2}} = \boldsymbol{vibration}) \quad (7)$$

In other words, button press behaviors were sampled from the posterior distribution over vibration vs. no vibration states, such that choices to press became more likely as the posterior probability of a vibration approached 1 and choices not to press became more likely as the posterior probability of a vibration approached 0.

Our model also considered the existence of learning processes, meaning that participants could update their prior beliefs about the probability of feeling a vibration vs. no vibration after each trial, based on how frequently they believed they had felt a vibration in the past. Essentially, every time a vibration is felt, prior beliefs favoring feeling a vibration go up, and every time that no vibration is felt this (relative) belief goes back down. Formally, this corresponds to updating the concentration parameters of Dirichlet (Dir) priors associated with the **B** matrix ($\boldsymbol{b}$) that specify beliefs about state transitions (Equation 8).

$$p(\boldsymbol{B}) = Dir(\boldsymbol{b})$$

$$pb = \begin{bmatrix} 0 & 0 & 0 \\ 1 - pV & 1 & 0 \\ pV & 0 & 1 \end{bmatrix} \quad (8)$$

$$\boldsymbol{b}_{trial} = pb + (1 - \omega) \cdot (\boldsymbol{b}_{trial-1} - pb) + \eta \cdot (S_{t=2} \otimes S_{t=1})$$

where $\otimes$ indicates the cross-product, and η_{pV} is a scalar that controls the magnitude of change in concentration parameters after each trial. The parameter η acts as a learning rate, controlling the size of the count added to the Dirichlet distribution after each observation. This parameter was estimated separately for vibration and no-vibration trials in the winning model. In some models in our model space, a similar learning process was tested for updating beliefs about IP over time, but this was not found to best account for the data.

Participant responses were used to fit a validated Bayesian model of interoceptive inference developed for this task.[12] In our model, uncertainty arises from multiple sources. First, sensory signals from the body are noisy and therefore only partially resolve uncertainty about the presence of vibration versus no-vibration states, and individuals may differ in how noisy they expect these signals to be. Second, there is uncertainty in how frequently vibrations will occur, with evolving priors over states that are learned through experience in the task. Finally, as vibration frequency is itself variable across the task, uncertainty is also present regarding what this frequency is. Consistent with this framework, participants infer the presence of a vibration on each 3-s trial based on expected signal precision (interoceptive precision), prior beliefs about state probabilities (frequency of presence vs. absence of vibration), and evolving confidence in these state probabilities, which adapts according to the frequency of felt vibrations over time on the task.

2. Fitting the model to behavioral data

© 2026 Verdonk C et al. *JAMA Psychiatry*.

Our method for parameter estimation utilized a commonly used Bayesian optimization algorithm, known as Variational Laplace,[13], to estimate the computational parameter values for each participant. This algorithm maximized the likelihood of participants' responses, assuming that the posterior probability of perceiving a capsule vibration could be identified with the likelihood of choosing to press the button. We optimized the parameters using this likelihood and variational Laplace,[13] through the *spm_nlsi_Newton.m* parameter estimation routine available in the SPM12 software package (Wellcome Trust Centre for Neuroimaging, London, UK, http://www.fil.ion.ucl.ac.uk/spm). This approach helps mitigate overfitting by imposing complexity cost on parameters that deviate significantly from their prior values.

Estimating parameters required setting prior means and prior variances for each parameter. The prior variance for each parameter was set at a high precision value of 1/4 to help prevent overfitting, and the prior means were assigned as follows (Table S3):

eTable 3. Prior means for key model parameters.

Parameter	Prior mean value
Interoceptive precision (IP)	0.95
Difference in interoceptive precision (IP_{diff})[§]	0.25
Initial prior beliefs (pV)	0.5
Learning rates (η_V and η_{NV})	0.5

[§] Reflects the drop in IP value from the enhanced-intensity to normal-intensity block

Our selection of these priors was informed by our previous study that introduced this approach.[12] The precision priors assume high precision for the high-intensity block and an intermediate level of precision (.95-.25=0.7) for normal-intensity block.

The prior values for initial prior beliefs (pV) and learning rates (η_V and η_{NV}) were specifically selected to reduce estimation bias, with pV = 0.5 reflecting flat initial prior beliefs, and η_V and η_{NV} equal to 0.5, avoiding any bias toward values near the extremes of 0 or 1.

3. Model comparison and computational parameter recoverability

We assessed the relative evidence for multiple models incorporating different parameter combinations (Table S4). In addition to the parameters described in the previous section (Table S3), these models included included variations in learning mechanisms (with different possible learning rates) for updating pV. After fitting parameters for each model, we conducted Bayesian model comparison to identify the best-fitting model.[11]
Once the optimal model was selected, we verified the recoverability of the associated parameters. Specifically, we simulated behavior using a range of parameter value combinations representative of each participant, estimated parameters from the simulated data, and assessed correlations between generative and estimated parameters. The high correlation between these values confirmed the robustness and recoverability of each parameter.

eTable 4. Overview of model variants and their corresponding parameters.

Parameter	IP	IP_{diff}	pV	η_{IP}	η_{pV}
Model 1	✓		✓		
Model 2	✓	✓	✓		
Model 3	✓		✓		✓
Model 4	✓	✓	✓		✓
Model 5	✓		✓		✓|✓
Model 6*	✓	✓	✓		✓|✓
Model 7	✓		✓	✓	
Model 8	✓		✓	✓	✓
Model 9	✓		✓	✓	✓|✓
Model 10	✓		✓	✓|✓	
Model 11	✓		✓	✓|✓	✓
Model 12	✓		✓	✓|✓	✓|✓

✓ indicates that the corresponding model includes the parameter represented by the respective column; ✓|✓ indicates that the learning rate was split, with separate learning rates for vibration and no-vibration trials; IP: interoceptive precision; IP_{diff}: difference in IP between normal- and enhanced-intensity stimulations; pV: initial prior beliefs; η_{IP}: learning rate for IP values; η_{pV}: learning rate for pV values; * indicates the winning model (see results below).

© 2026 Verdonk C et al. *JAMA Psychiatry*.

eMethods 5. Processing of peripheral physiological data

1. Electrogastrogram data

The single-channel electrogastrographic (EGG) data were processed using custom MATLAB scripts (MATLAB 2021a, MathWorks®) in combination with functions from the FieldTrip toolbox (version 20171022[14]) and publicly available code developed by Wolpert et al. (2020) (https://github.com/niwolpert/EGG_Scripts/tree/master[15]).
The raw EGG signal, originally recorded at 1000 Hz, was downsampled to 250 Hz. It was then bandpass-filtered around the dominant gastric frequency using Wolpert's *compute_filter_EGG()* function, which applies a third-order finite impulse response (FIR) filter with a bandwidth of ±0.015 Hz. Artifact rejection followed criteria defined by Wolpert et al. (2020), whereby cycles exceeding ±3 standard deviations of the cycle length distribution or exhibiting nonmonotonic phase changes were identified and removed.[15] Spectral analysis was conducted using a Fast Fourier Transform (FFT) implemented in the *ft_freqanalysis()* function of the Fireldtrip toolbox to extract key features of gastric motility. To quantify gastric activity, absolute power was calculated for four frequency bands, consistent with our previous work[3]: normogastria (2.5–3.5 cpm), tachygastria (3.75–9.75 cpm), bradygastria (0.5–2.25 cpm), and total power (0.5–11 cpm).

2. Cardiac data

The electrocardiogram (ECG) data were processed using custom MATLAB scripts (MATLAB 2021a, MathWorks®) in conjunction with the FieldTrip toolbox (version 20171022[14]).
The raw ECG signal, initially recorded at 1000 Hz, was downsampled to 250 Hz. Interbeat intervals were extracted from R-peak detection, which was performed using custom scripts incorporating MATLAB's *findpeaks()* function. Consistent with our prior research,[3] we quantified heart rate variability using the following indices: (1) The standard deviation of R–R intervals (SDNN), a widely used measure of overall heart rate (HR) variability, which has been proposed to reflect autonomic regulation of HR;[16] (2) The phasic-HR, which captures transient heart rate changes induced by capsule vibration. For each period, it was calculated as the difference in HR between the 3-second (s) vibratory stimulation period and the 3-s pre-stimulation period (i.e., the 3 seconds immediately preceding vibration onset). To facilitate baseline comparisons, 60 pseudo-vibration onsets (each of 3-second duration) were introduced into the 30-minute baseline recording, with the first 2 minutes of the baseline excluded to ensure a physiological steady state; and (3) The tonic-HR, also referred to as resting HR, was calculated by averaging HR over 60-s windows.

3. Skin conductance data

Skin conductance data were processed using custom MATLAB scripts (MATLAB 2021a, MathWorks®) alongside the Ledalab Toolbox (version 3.49[17]). The raw SCR signal was recorded at 1000 Hz and downsampled to 25 Hz. MATLAB's *detrend()* function was then applied to remove slow signal drifts. Phasic changes in skin conductance responses (SCRs) to capsule stimulation were analyzed using Continuous Deconvolution Analysis.[17] Following our previous work[3], we quantified SCRs as the logarithmic transformation of the maximum phasic activity during the 3-second vibration period, relative to the 3-second pre-stimulus period, applying a 0.01 micro Siemen (µS) threshold.[18,19] To assess SCR variations relative to resting physiological conditions, we used the same pseudo-events from the baseline period, as in the cardiac data analysis (Supplementary Methods 5. Processing of peripheral physiological data).

© 2026 Verdonk C et al. *JAMA Psychiatry*.

eMethods 6. Illness status definitions

Illness status was assessed remotely at the 6-month follow-up using an operationalized definition based on our previously proposed standardized criteria.[20] This standardized definition incorporates self-reported symptoms, behaviors, Body Mass Index (BMI), and duration.

1. **Symptoms**

The behavior of eating restriction was assessed using responses on the Restraint subscale of the Eating Disorder Inventory-3 (EDI-3), which evaluates preoccupation with weight, excessive dieting concerns, and fear of gaining weight.[21] Symptom severity was categorized as follows: a score of ≤ 7 indicated non-significant fear (corresponding to an average response of "Never" or "Rarely"), a score > 7 and ≤ 21 indicated the presence of fear (average response of "Sometimes" or "Often"), and a score > 21 indicated significant fear (average response greater than "Often").

2. **Behaviors**

The behavior of restricting was assessed using the Restraint subscale of the Eating Disorder Examination Questionnaire (EDE-Q).[22] Severity was categorized as follows: a score of ≤ 2 indicated non-significant restriction (occurring 5 or fewer days per month), a score > 2 and ≤ 4 indicated the presence of restriction (occurring more than 5 but no more than 22 days per month), and a score > 4 indicated significant restriction (occurring more than 22 days per month).
Similarly, bingeing and purging behaviors were evaluated using the Bulimia subscale of the Eating Disorder Inventory-3 (EDI-3).[21] Scores were interpreted as follows: ≤ 8 indicated non-significant bingeing/purging ("Never" or "Rarely"), > 8 and ≤ 24 indicated the presence of bingeing/purging ("Sometimes" or "Often"), and > 24 indicated significant bingeing/purging (more frequent than "Often").
Purging behaviors involving vomiting or laxative use were further examined using items 26 and 32 from the Body Shape Questionnaire (BSQ).[23] A score of 1 ("Never") indicated non-significant purging, a score of 2 or 3 ("Rarely" or "Sometimes") indicated the presence of purging, and a score ≥ 4 ("Often") indicated significant purging.
Finally, excessive exercise as a purging behavior was assessed using item 4 of the Exercise Addiction Inventory (EAI).[24] Scores were categorized as follows: ≤ 3 indicated no increase in exercise, a score of 4 indicated a present increase, and a score of 5 indicated a significant increase in exercise over time.

3. **Weight and shape concerns**

Weight and shape concerns were evaluated using responses on the Weight Concern and Shape Concern subscales of the EDE-Q. These subscales were selected to avoid overemphasizing or duplicating the Restraint subscale, which was separately operationalized to define restricting behaviors. Specifically, average subscale scores within 1.5 standard deviations (SD) of the HC sample were considered indicative of partial recovery (in combination with the aforementioned symptom and behavior score cutoffs). Scores within 2 SD suggested either full or partial remission, while scores ≥ 2 SD were consistent with partial or full relapse.

4. **Body Mass Index**

BMI was calculated using self-reported height and weight data collected through the EDE-Q. While we asked participants to provide this information, it was not a requirement of the remote follow-up data collection. At the 6-month follow-up, 87% of participants self-reported the data necessary for BMI computation.

Based on these definitions, each AN participant's illness status at 6 months was classified as one of the following:

Full relapse: defined as the presence of significant fear of weight gain (EDI-3 Drive for Thinness subscale score > 21), significant restricting behaviors (EDE-Q Restraint subscale score > 4), the presence of bingeing and/or purging behaviors (EDI-3 Bulimia subscale score > 24, BSQ items 26 and 32 scored > 3, and EAI item 4 scored > 4), EDE-Q Weight Concern and Shape Concern subscale averages ≥ 2 SD above those of the HC sample, and a BMI ≤ 18.5.

Partial relapse: defined as the presence of significant fear of weight gain (EDI-3 Drive for Thinness subscale score > 21), the presence of restricting behaviors (EDE-Q Restraint subscale score > 2 and ≤ 4), bingeing and/or purging behaviors (EDI-3 Bulimia subscale score > 8 and ≤ 24, BSQ items 26 and 32 scored ≤ 3, and EAI item 4 scored ≤ 4),

© 2026 Verdonk C et al. *JAMA Psychiatry*.

EDE-Q Weight Concern and Shape Concern subscale averages ≥ 2 SD above those of the HC sample, and a BMI ≤ 18.5.

Partial remission: defined using the same criteria as full remission, with the exception that the BMI requirement was ≥ 18.5 but < 19.

Full remission: defined as the presence of fear of weight gain (EDI-3 Drive for Thinness subscale score > 7 and ≤ 21), but without restricting behaviors (EDE-Q Restraint subscale score ≤ 2), bingeing or purging behaviors (EDI-3 Bulimia subscale score ≤ 8, BSQ items 26 and 32 scored as 1, and EAI item 4 score ≤ 3). Additionally, individual with AN needed to have EDE-Q Weight Concern and Shape Concern subscale averages within 2 SD of the healthy control (HC) sample and a BMI ≥ 19.

Partial recovery: defined as the absence of significant fear of weight gain (EDI-3 Drive for Thinness subscale score ≤ 7), no restricting behaviors (EDE-Q Restraint subscale score ≤ 2), no bingeing or purging behaviors (EDI-3 Bulimia subscale score ≤ 8, BSQ items 26 and 32 scored as 1, and EAI item 4 score ≤ 3), EDE-Q Weight Concern and Shape Concern subscale averages within 1.5 SD of the HC sample, and a BMI ≥ 19.

Full recovery: this was not included as a possible 6-month outcome due to the duration requirement of 1 year for this classification.

© 2026 Verdonk C et al. *JAMA Psychiatry*.

eMethods 7. Statistical analysis

1. Behavioral, self-report, computational, and peripheral physiological data

To assess whether vibratory capsule-induced signals influenced behavioral, self-report, computational, and peripheral physiological data, we employed linear mixed-effects (LME) models. These models evaluated the effects of diagnostic group (AN vs. HC), block (normal vs. enhanced), and period (baseline, normal, enhanced) on the dependent variables. LME models included diagnostic group, block or period, age, and BMI as fixed effects, with participant modeled as a random factor, and measures of GI interoception and peripheral physiological measures as dependent variables. Interactions between diagnostic group and block or period were explicitly tested. To reduce the risk of Type I errors, models were estimated using Restricted Maximum Likelihood (REML), and p-values were derived via Type II analysis of variance (ANOVA) with Satterthwaite's method.[25,26] Post-hoc analyses were conducted using estimated marginal means, with Kenward-Roger degrees of freedom.[27] The confidence levels and p values were adjusted using a mvt method. Effect size estimates are reported as partial Eta squared (η_p^2) for Type II ANOVA and as Cohen's d for post-hoc tests.

We also employed linear models to examine whether computational measures were influenced by capsule vibration-induced changes in gastric signals, considering diagnostic group as a key factor. These measures were calculated across both blocks, with age and BMI included as covariates.

Statistical analyses were conducted in R (version 4.2.1[28]) using the *lme4* [29], *lmer* [30], and *emmeans* packages.

2. Electroencephalographic data

We applied non-parametric cluster permutation testing to examine whether gut mechanosensory stimulations induced by capsule vibration influenced vibration-elicited gastric-evoked potential (GEP) amplitudes as a function of diagnostic group (AN vs. HC). This data-driven statistical method all GEP owed for group comparisons across all electrodes and time points, offering a robust approach to handling the multiple comparison problem.[31,32] The underlying assumption of this approach is that a biologically meaningful effect should exhibit spatial and temporal coherence across neighboring electrodes and time points. Statistical significance was determined by comparing the observed statistical results with a distribution of values generated through permutation-based shuffling of the data.[31,32] Given the absence of prior assumptions regarding the spatial and temporal distribution of GEP differences between diagnostic groups, this exploratory approach was particularly well suited to our study.

Cluster identification involved the following steps: (a) t-statistics were calculated for each sample in the spatiotemporal GEP data to compare diagnostic groups (AN vs. HC); (b) these statistics were thresholded by a p-value ($p < 0.05$); (c) neighboring data points that exceeded the threshold and had the same sign were grouped; (d) cluster-level statistics were obtained by selecting the maximum t-statistic within each cluster; and (e) the maximum cluster statistic was assessed against its permutation distribution. This permutation distribution was derived from statistical values of independent t-tests performed on 10,000 random permutations of GEP data relative to diagnostic group, with a p-value threshold of 0.05 for cluster inclusion.[33] The analysis accounted for both spatial (electrodes) and temporal (time points) dimensions. Electrodes within 2.5 cm were considered neighbors, averaging 5.4 neighbors per electrode, and a minimum of two neighboring electrodes was required for a sample to be included in the clustering process.

Group differences (AN versus HC) were analyzed separately within each block (normal and enhanced). Cluster permutation was conducted within the whole vibration time window (-200 ms to +3000 ms) using custom scripts in Matlab 2021a and the Fieldtrip toolbox (version 20171022[14]).

Average GEP amplitude (avGEP) was calculated across midline parieto-occipital electrodes (P3, P4, O1, O2, Pz, Oz, CP1, CP2, and POz) and across time points (364 to 740 ms following vibration onset). This spatiotemporal window was identified in the present study as the common EEG marker of gut mechanosensation across diagnostic groups (AN and HCs; see Figures 3A-B), and is consistent with the spatiotemporal electrode findings reported in our previous work15. We then explored the relationship between neural, behavioral, computational, physiological, and subjective outcomes to examine multilevel associations of interoceptive processing across diagnostic groups using Spearman correlation analyses.

3. Longitudinal analysis

In the AN group, logistic and linear regressions evaluated whether pre-discharge task parameters predicted 6-month relapse outcomes or global scores on the Eating Disorder Examination Questionnaire (EDE-Q), a standard and widely accepted tool for capturing global eating disorder severity.[34] Illness status at 6 months was assessed remotely using

© 2026 Verdonk C et al. *JAMA Psychiatry*.

operational criteria adapted from our previous standardized definitions,[20] which classified participants as Full relapse, Partial relapse, Partial remission, Full remission, Partial recovery, or Full recovery (Supplementary Methods 6. Illness status definitions). For the logistic models, categories were dichotomized as "Full relapse" vs "Not full relapse" (aggregating Partial relapse, Partial remission, Full remission, Partial recovery, and Full recovery) to address limited variance and ensure stable estimation. Predictors in the logistic regression were standardized by dividing their values by two within-group standard deviations.[35] No scaling was applied in the linear models. Age and BMI were included as covariates in all predictive models.

© 2026 Verdonk C et al. *JAMA Psychiatry*.

eResults 1. Behavioral and self-reported findings

1. Behavioral findings

Regardless of group, normal stimulation was associated with decreased perceptual accuracy (AN: $P<0.001$, $d=-2.22$; HCs: $P<0.001$, $d=-1.43$), increased miss rates (AN: $P<0.001$, $d=2.21$; HCs: $P<0.001$, $d=1.02$), and a greater tendency not to press the button (i.e., response bias), with a significant effect in HCs ($P=0.03$, $d=-0.61$) but not in AN ($P=0.10$, $d=-0.42$).

2. Self-report findings

2.1. Interoceptive intensity

For stomach/digestive sensations, intensity increased during the task ($P<0.001$; $\eta^2_p=0.49$), but surprisingly, it did not differ by group ($P=0.73$) (eFigure 2A). For breathing sensations, there was a main effect of group ($P<0.001$, $\eta^2_p=0.14$; eFigure 2B), with AN individuals reporting greater breathing sensation intensity than HCs both before ($P=0.007$, Cohen's $d=0.65$) and during the task ($P<0.001$, Cohen's $d=1.03$). For heartbeat sensations, the group-by-time interaction was significant ($P=0.004$, $\eta^2_p=0.07$; eFigure 2C). Post-hoc comparisons indicated that AN participants reported a significant increase from before to during the task ($P<0.001$, Cohen's $d=0.86$), whereas HCs did not ($P=0.61$). For muscle tension, there was a main effect of group ($P<0.001$, $\eta^2_p=0.13$; eFigure 2D), with AN individuals reporting greater muscle tension than HCs both before ($P<0.001$, Cohen's $d=1.03$) and during the task ($P=0.01$, Cohen's $d=0.70$).

2.2. Interoceptive valence

Group differences in valence were marginal for stomach/digestive ($P=0.06$; eFigure 2E) and breathing sensations ($P=0.07$; eFigure 2F), and non-significant for heartbeat sensations ($P=0.24$; eFigure 2G). No significant effects of time or group-by-time interactions were observed for any of the valence measures.

2.3. Homeostatic interoceptive urges

Thirst decreased after the task in both groups ($P<0.001$; $\eta^2_p=0.28$; eFigure 2I) and was consistently lower in the AN group than in HCs ($P<0.001$; $\eta^2_p=0.05$). The urge to urinate increased after the task in both groups ($P<0.001$; $\eta^2_p=0.36$; eFigure 2J) and was higher in AN individuals than in HCs ($P=0.05$; $\eta^2_p=0.03$). Urge to defecate likewise increased in both groups after the task ($P<0.001$; $\eta^2_p=0.17$; eFigure 2K) and was higher in the AN group than in HCs ($P=0.04$; $\eta^2_p=0.04$).

© 2026 Verdonk C et al. *JAMA Psychiatry*.

eFigure 2. **Retrospective ratings of interoceptive sensations and homeostatic urges before, during, and after vibratory gut stimulation.**

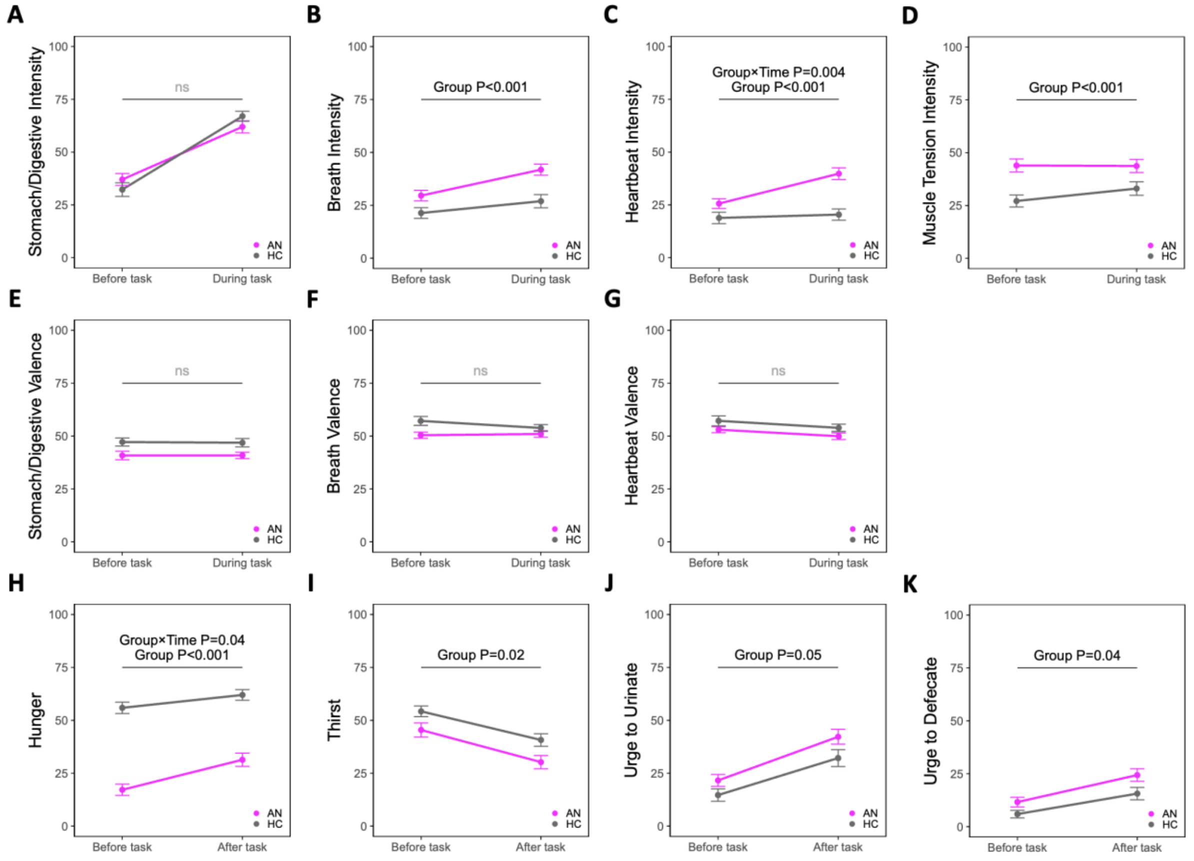

(A) Stomach/digestive intensity: increased during the task in both groups, with no group differences. **(B)** Breathing intensity: higher in individuals with AN than HCs both before and during the task. **(C)** Heartbeat intensity: group by time interaction, with an increase during the task in AN but no change in HCs. **(D)** Muscle tension: higher in AN than HC both before and during the task. **(E–G)** Valence: marginal group differences for stomach/digestive and breathing; none for heartbeat. **(H)** Hunger: group by time interaction, with a larger task-associated increases in the AN group. **(I)** Thirst: decreased during the task in both groups and was consistently lower in the AN group than HC. **(J–K)** Urges to urinate/defecate: increased from pre to post in both groups and were higher in AN than in HC. **Note**: Main effects of time were observed for Stomach, Breath, Heartbeat intensity (A–C), Heartbeat valence (G), and Hunger, Thirst, Urination and Defecation urge (H–K) but are not displayed (see results for details). Muscle tension valence was not assessed. Abbreviations: AN, anorexia nervosa; HC, healthy comparison.

© 2026 Verdonk C et al. *JAMA Psychiatry*.

eResults 2. Electroencephalogram findings

1. Group comparisons of onset-evoked GEP responses

Cluster-based permutation testing did not reveal significant group differences in vibration onset-evoked GEP amplitude across blocks (normal block: Monte Carlo P>0.27; enhanced block: Monte Carlo P>0.13; eFigure 3).

eFigure 3. Group overlay of onset-evoked gastric-evoked potential (GEP) responses.

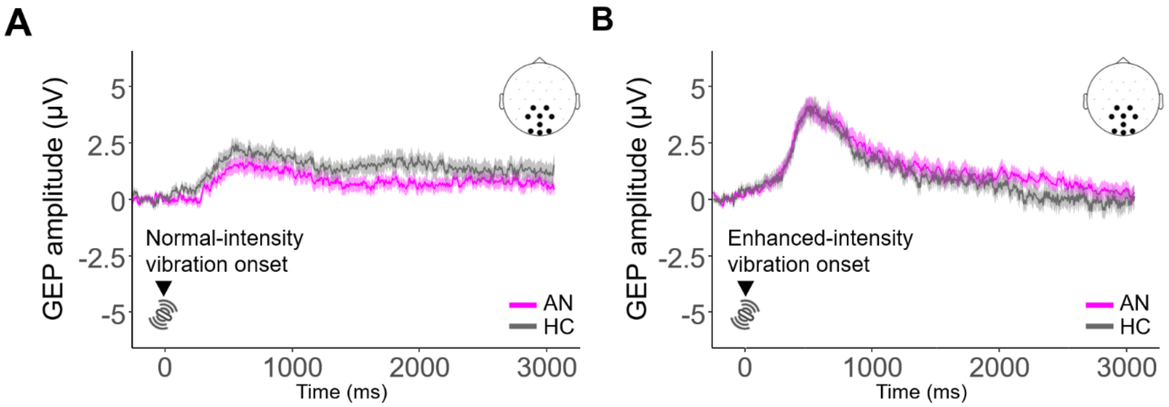

There were no significant differences in GEP amplitude between the AN and HC groups during the normal block **(A)** and the enhanced block **(B)**. Error bars indicate standard error of the mean. *Note: The GEP was computed from the cluster of centro-right parieto-occipital electrodes exhibiting a significant intensity-vibration (block) effect in both groups.* AN – anorexia nervosa, HC – healthy comparison.

2. Group comparisons of offset-evoked GEP responses

We did not find significant group differences in GEP amplitude across blocks (normal block: Monte Carlo P>0.20; enhanced block: Monte Carlo P>0.34; eFigure 4). However, enhanced stimulations elicited significantly larger GEP amplitudes compared to normal stimulation in both groups.

© 2026 Verdonk C et al. *JAMA Psychiatry*.

eFigure 4. Group overlay of offset-evoked gastric-evoked potential (GEP) responses.

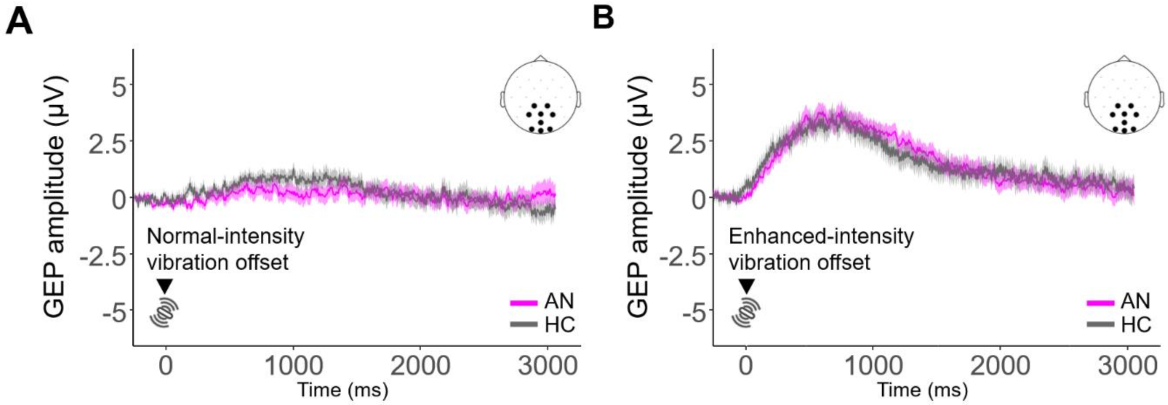

GEP amplitudes did not differ significantly between the AN and HC groups during the normal block **(A)** or the enhanced block **(B)**. Error bars indicate standard error of the mean. *Note: The GEP was computed from the cluster of centro-right parieto-occipital electrodes exhibiting a significant intensity-vibration (block) effect in both groups.* AN – anorexia nervosa, HC – healthy comparison.

© 2026 Verdonk C et al. *JAMA Psychiatry*.

eResults 3. Computational findings

1. Model comparison and parameter recoverability

Model comparison confirmed that a model with each of the five parameters mentioned above (Model 6; see Table S4 in Supplementary Methods 4) provided the best fit to the data, with a protected exceedance probability of 1 (eTable 5). This model outperformed several alternative models that included different subsets of these and other possible parameters.

eTable 5. Model comparison results.

	Protected exceedance probability
Model 1	4.09×10^{-43}
Model 2	4.09×10^{-43}
Model 3	4.09×10^{-43}
Model 4	4.09×10^{-43}
Model 5	4.09×10^{-43}
Model 6	1
Model 7	4.09×10^{-43}
Model 8	4.09×10^{-43}
Model 9	4.09×10^{-43}
Model 10	4.09×10^{-43}
Model 11	4.09×10^{-43}
Model 12	4.09×10^{-43}

Recoverability analyses confirmed that these parameters were reliably recoverable within the range of values observed in participant estimates. Specifically, when generating simulated behavior using the parameter value combinations observed in participant estimates and then re-estimating the parameters from the simulated data, the correlations between true and estimated parameters were strongly positive (rs > 0.77; Ps<0.001; eTable 6).

eTable 6. Correlation results for parameter recoverability analysis.

Parameter	Correlation between true and estimated parameters	
	r	P-value
Interoceptive precision (IP)	0.91	<0.001
Difference in interoceptive precision (IP_{diff})	0.97	<0.001
Initial prior beliefs (pV)	0.64	<0.001
Learning rate for trials with capsule vibration (η_V)	0.77	<0.001
Learning rate for trials without capsule vibration (η_{NV})	0.92	<0.001

2. Exploratory visualization of prior time-course

The combination of group differences in initial prior beliefs (Figure 4A) and learning rate (Figure 4C) may have contributed to between-group differences in the time course of prior beliefs: based on visual inspection, the AN group showed a stronger drift in prior values toward not detecting a vibration as the task progressed (eFigure 5).

eFigure 5. Prior time-course.

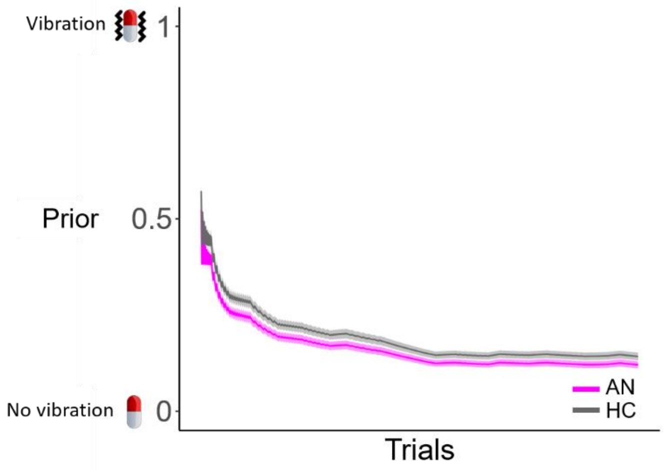

Group-level time course of prior belief values across trials illustrates that the AN group showed a stronger drift below the neutral 0.5 line, indicating a stronger bias against detecting a vibration as the task progressed. Abbreviations: AN, anorexia nervosa; HC, healthy comparison.

© 2026 Verdonk C et al. *JAMA Psychiatry*.

eResults 4. Longitudinal findings

At the 6-month follow-up, 16 AN individuals were classified as fully relapsed, while 38 were classified under another AN-related condition including partial recovery, full remission, partial remission, or partial relapse (see Supplementary Methods 6 and eFigure 6). 6-month data were missing for 8 AN individuals due to dropout.

eFigure 6. Individual trajectories of eating disorder symptoms from discharge to 6-month follow-up in an.

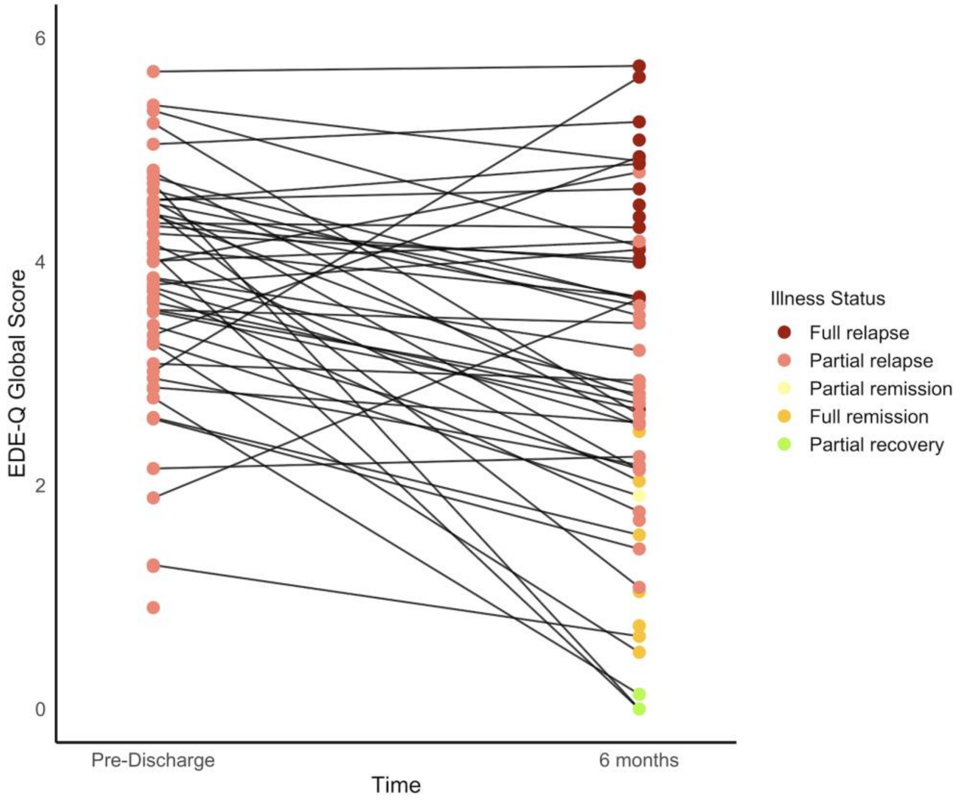

Spaghetti plot of AN participants showing eating disorder symptoms (indexed via the EDE-Q Global Score) at the baseline pre-discharge visit and again at the 6-month follow-up visit. Lower EDE-Q Global Scores indicate lower eating disorder symptomatology. The color coding of each individual's clinical status at each visit timepoint corresponds to the operationalized definitions for relapse, remission and partial recovery adapted from our previously proposed standardized criteria [20].

1. **Relapse outcome at 6 months**

At 6 months, relapse outcome was successfully predicted by experimental session measures. Specifically, initial prior beliefs (pV) (Odds Ratio (OR)=3.82; P=0.05) and response bias in the normal block (OR=5.37; P=0.04) predicted relapse, such that AN individuals with stronger initial prior beliefs against perceiving GI signals, as reflected by lower pV values, were more likely to be fully relapsed at six months. Similarly, AN individuals with more positive response bias values, meaning they were less likely to press the button during normal stimulations, were also more likely to be in full relapse at six months (eTable 7). In addition, higher stomach unpleasantness during the task was associated with greater odds of full relapse (OR=5.73, P=0.03; eTable 8).

eTable 7. Logistic regression results predicting 6-month AN status (full relapse) from experimental session measures. Statistically significant predictions are bolded, with the corresponding Odd Ratio (OR) values reported.

**Predictor**	**Outcome 6-month AN status**	
	P-value	OR
Interoceptive precision (IP)	0.20	-
Difference in interoceptive precision (IP_{diff})	0.10	-
Initial prior beliefs (pV)	**0.05**	**3.82**
Learning rate for trials with capsule vibration (η_V)	0.71	-
Learning rate for trials without capsule vibration (η_{NV})	0.45	-
Normalized A’ (Normal block)	0.77	-
Normalized A’ (Enhanced block)	0.74	-
Miss rate (Normal block)	0.64	-
Miss rate (Enhanced block)	0.36	-
Response bias (Normal block)	**0.04**	**5.37**
Response bias (Enhanced block)	0.94	-
Response time (Normal block)	0.18	-
Response time (Enhanced block)	0.82	-

© 2026 Verdonk C et al. *JAMA Psychiatry*.

eTable 8. Self-report measures: logistic regression predicting 6-month AN illness status (full relapse) from retrospective ratings of sensation intensity and valence (unpleasant/pleasant) and state items before, during, and after vibratory gut stimulation. Statistically significant predictions are bolded, with the corresponding Odds Ratio (OR) values reported.

Predictor	Outcome<br>6-month full relapse status	
	P-value	OR
**Intensity of sensations**	*Before* | *During*	*Before* | *During*
Stomach/digestive	0.10 | 0.55	- | -
Breath	0.11 | 0.48	- | -
Heartbeat	0.21 | 0.49	- | -
Muscle tension	0.69 | 0.81	- | -
**Valence of sensations**	*Before* | *During*	*Before* | *During*
Stomach/digestive	0.38 | **0.03**	- | **5.73**
Breath	0.87 | 0.67	- | -
Heartbeat	0.48 | 0.43	- | -
**State**	*Before* | *After*	*Before* | *After*
Hunger	0.19 | 0.49	- | -
Thirst	0.21 | 0.22	- | -
Urge to urinate	0.31 | 0.90	- | -
Urge to defecate	0.10 | 0.62	- | -

2. Eating disorder symptom severity at 6 months

At 6 months, eating disorder symptom severity, indexed by EDE-Q scores, was also significantly predicted by several experimental session measures, including miss rate (P=0.05; adjusted R squared (R^2)=0.08), difference in IP between normal and enhanced stimulations (IP_{diff}; P=0.004; R^2=0.16), and initial prior beliefs (pV; P=0.05; R^2=0.09) (eTable 9). In addition, Eating disorder symptom severity at 6 months was predicted by stomach valence ratings before (P=0.02, R^2=0.11) and during the task (P=0.02, R^2=0.11; eTable 10). However, none of the homeostatic interoceptive urges items were predictive of relapse outcome or EDE-Q score at 6 months.

Information on the total length of treatment stay was not directly available, as discharge dates were not collected as part of the study. However, we approximated treatment duration by calculating the interval between admission and the experimental session, given that discharge typically occurred within the following 7–10 days. Based on this approximation, mean treatment stay at the time of testing was approximately 59 ± 24 days. Treatment duration was not significantly associated with any behavioral, computational, or eating disorder symptom severity measures (e.g., EDE-Q); further, the r values were all quite low (e.g., r's from 0.03 to 0.21).

© 2026 Verdonk C et al. *JAMA Psychiatry*.

eTable 9. Linear regression results predicting EDE-Q Total score at 6 months from experimental session measures. Statistically significant predictions are bolded, with the corresponding regression coefficient (β) and adjusted R squared (R^2) values reported.

**Predictor**	**Outcome**		
	**6-month EDE-Q Total score**		
	P-value	β	R^2
Interoceptive precision (IP)	0.61	-	-
Difference in interoceptive precision (IP_{diff})	**0.004**	**5.84**	**0.16**
Initial prior beliefs (pV)	**0.05**	**-2.99**	**0.09**
Learning rate for trials with capsule vibration (η_V)	0.06	-	-
Learning rate for trials without capsule vibration (η_{NV})	0.25	-	-
Normalized A' (Normal block)	0.23	-	-
Normalized A' (Enhanced block)	0.29	-	-
Miss rate (Normal block)	**0.05**	**1.05**	**0.08**
Miss rate (Enhanced block)	0.46	-	-
Response bias (Normal block)	0.14	-	-
Response bias (Enhanced block)	0.80	-	-
Response time (Normal block)	0.53	-	-
Response time (Enhanced block)	0.47	-	-

© 2026 Verdonk C et al. *JAMA Psychiatry*.

eTable 10. Self-report measures: linear regression results predicting EDE-Q Total score at 6 months from retrospective ratings of sensation intensity and valence (unpleasant/pleasant) and state items before, during, and after vibratory gut stimulation. Statistically significant predictions are bolded, with the corresponding regression coefficient (β) and adjusted R squared (R^2) values reported.

**Predictor**	**Outcome**		
	**6-month EDE-Q Total score**		
	P-value	β	R^2
**Intensity of sensations**	*Before* | *During*	*Before* | *During*	*Before* | *During*
Stomach/digestive	**0.01** | 0.83	**0.02** | -	**0.13** | -
Breath	0.17 | 0.48	- | -	- | -
Heartbeat	0.16 | 0.97	- | -	- | -
Muscle tension	0.81 | 0.09	- | -	- | -
**Valence of sensations**	*Before* | *During*	*Before* | *During*	*Before* | *During*
Stomach/digestive	**0.02** | **0.02**	**-0.03** | **-0.04**	**0.11** | **0.11**
Breath	0.67 | 0.73	- | -	- | -
Heartbeat	0.97 | 0.34	- | -	- | -
**State**	*Before* | *After*	*Before* | *After*	*Before* | *After*
Hunger	0.73 | 0.62	- | -	- | -
Thirst	0.50 | 0.45	- | -	- | -
Urge to urinate	0.71 | 0.97	- | -	- | -
Urge to defecate	0.28 | 0.48	- | -	- | -

© 2026 Verdonk C et al. *JAMA Psychiatry*.

eResults 5. Peripheral physiological findings

Compared to HCs, the AN group exhibited a significantly lower tonic (i.e., basal) heart rate (HR) across both the baseline period and stimulation blocks ($P<0.001$, $\eta^2_p=0.37$; eFigure 7A). These group differences were significantly more pronounced during the stimulation blocks compared to baseline (period-by-group interaction effect: $P=0.05$, $\eta^2_p=0.03$). However, no significant group differences were observed for phasic HR activity (i.e., rapid responses specific to the 3-second vibration period; P=0.72) or heart rate variability (P=0.17), as measured using the standard deviation of R–R intervals (SDNN) (eFigure 7B-C). Further, no significant group differences were observed for electrogastrogram (EGG) responses (eFigure 7D-E-F-G), including the absolute power across four gastric frequency ranges: total power (0.5–11 cpm; P=0.85), bradygastria (0.5–2.25 cpm; P=0.88), normogastria (2.5–3.5 cpm; P=0.86), and tachygastria (3.75–9.75 cpm; P=0.47). Finally, no group differences were observed in skin conductance response (SCR), specifically in the maximum value of phasic activity (P=0.97; eFigure 7H).

© 2026 Verdonk C et al. *JAMA Psychiatry*.

eFigure 7. Peripheral physiological responses during baseline and vibratory gut stimulation.

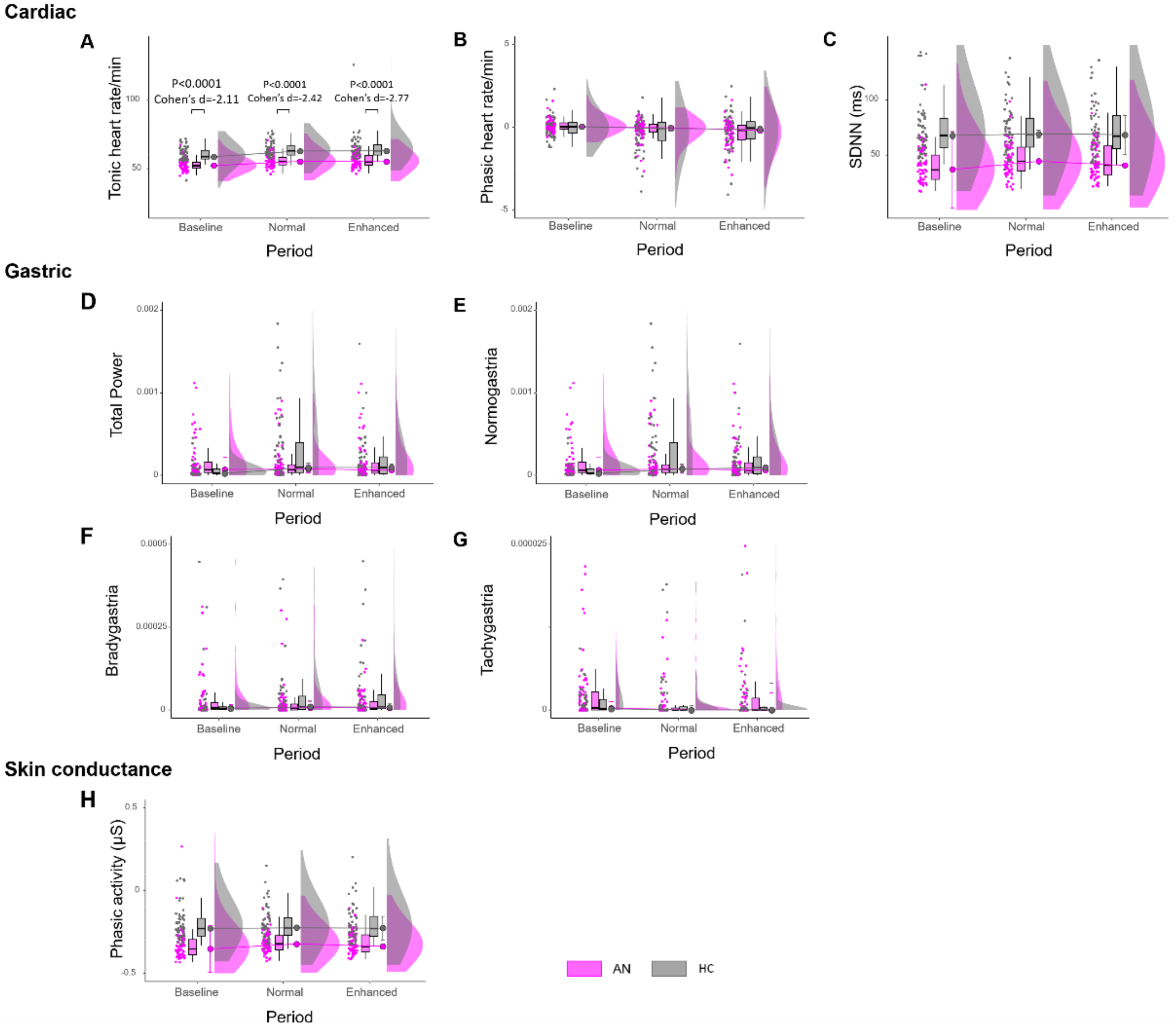

(A) The AN group exhibited a significantly lower tonic (basal) heart rate (HR) compared to HCs, with group differences becoming more pronounced during the stimulation periods relative to baseline. **(B-C)** No significant differences were observed between groups in phasic HR activity or heart rate variability, assessed via the standard deviation of R–R intervals (SDNN). **(D-E-F-G)**. Similarly, no group differences were found in electrogastrogram (EGG) responses, including absolute power across four gastric frequency bands: total power ([0.5–11] cpm), bradygastria ([0.5–2.25] cpm), normogastria ([2.5–3.5] cpm), and tachygastria ([3.75–9.75] cpm). **(H)** Skin conductance response (SCR) also showed no significant differences between groups, particularly in the maximum value of phasic activity. The plots display the distribution of individual data points, corresponding boxplots, and individual participant values. AN – Anorexia Nervosa, HC – healthy comparison, ms – milliseconds, µS – microsiemens. *Note: Only significant main group effects are reported in the figures.*

© 2026 Verdonk C et al. *JAMA Psychiatry*.

eResults 6. Multilevel correlation findings

The average GEP amplitude, derived from the spatiotemporal window identified as the electroencephalographic marker of gut mechanosensation (see Figures 3A-B in the main text), was significantly correlated with perceptual accuracy measures during the normal stimulation block and also showed associations with several computational parameters (eTable 11). Across all of these variables, correlations were consistently larger in the AN group, with higher absolute correlation coefficients and greater statistical significance compared to HCs.

© 2026 Verdonk C et al. *JAMA Psychiatry*.

eTable 11. Correlations between average gastric-evoked potential (avGEP) amplitude and behavioral or computational measures for the normal and enhanced stimulation blocks. Statistically significant correlations (p<0.05), following Holm correction, are bolded. *Note: The avGEP amplitude was derived from the spatiotemporal window identified as the electroencephalographic marker of gut mechanosensation across diagnostic groups (individuals with anorexia nervosa (AN; n=62) and healthy comparisons (HCs; n=57); see Figures 3A-B in the main text).*

	Correlation with the avGEP amplitude			
	Normal		Enhanced	
**Measure**	**AN**	**HC**	**AN**	**HC**
Normalized A’	***r*=0.70**<br>**p<0.001**	***r*=0.49**<br>**p=0.04**	*r*=0.20<br>p=1	*r*=0.33<br>p=1
Miss rate	***r*=-0.66**<br>**p<0.001**	***r*=-0.57**<br>**p<0.001**	*r*=-0.21<br>p=1	*r*=-0.34<br>p=1
Response time	***r*=-0.60**<br>**p=0.002**	***r*=-0.68**<br>**p<0.001**	*r*=-0.08<br>p=1	*r*=-0.34<br>p=1
Response bias	*r*=-0.01<br>p=1	*r*=-0.16<br>p=1	*r*=-0.15<br>p=1	*r*=-0.27<br>p=1
Initial prior beliefs (pV)	*r*=0.26<br>p=1	*r*=0.27<br>p=1	*r*=0.07<br>p=1	*r*=0.01<br>p=1
Interoceptive precision (IP)	*r*=0.27<br>p=0.11	*r*=0.11<br>p=1	*r*=0.21<br>p=1	*r*=0.36<br>p=1
Difference in interoceptive precision (IP_{diff})	***r*=-0.62**<br>**p<0.001**	*r*=-0.37<br>p=0.69	*r*=-0.19<br>p=1	*r*=-0.14<br>p=1
Learning rate for trials with capsule vibration (η_V)	***r*=0.58**<br>**p<0.001**	***r*=0.49**<br>**p=0.01**	*r*=0.37<br>p=0.36	*r*=0.22<br>p=1
Learning rate for trials without capsule vibration (η_{NV})	***r*=-0.60**<br>**p<0.01**	***r*=-0.46**<br>**p=0.04**	*r*=-0.38<br>p=0.34	*r*=-0.25<br>p=1

© 2026 Verdonk C et al. *JAMA Psychiatry*.

eDiscussion

1. Behavioral findings

The behavioral findings of the present study offer direct empirical support for disrupted GI interoception in weight-restored individuals with AN, evidenced by reduced interoceptive accuracy in detecting mechanosensory gut signals. Importantly, performance in the Enhanced condition was comparable across groups, suggesting that interoceptive deficits in AN may be attenuated when afferent signal strength is increased. This points to the potential utility of scalable interoceptive training interventions that use heightened bodily cues to support perceptual learning and improve internal signal tracking in clinical populations. Notably, the substantial inter-individual variability (especially within the AN group) suggests there is meaningful heterogeneity in GI interoceptive processing, which may have implications for personalized risk stratification and treatment targeting. For example, future studies could examine whether individuals with the greatest interoceptive impairment are most likely to benefit from interoceptive feedback training or targeted gut-focused interventions.

2. Peripheral physiological findings

Multimodal physiological assessment revealed selectively altered cardiac function in AN, specifically lower tonic heart rate during rest and stimulation, consistent with a shift in autonomic balance toward increased parasympathetic (vagal) activity following weight restoration.[36,37] In contrast to prior reports, we observed no significant group differences in heart rate variability, electrogastrogram, or skin conductance responses. These discrepancies may reflect methodological differences, including the short-term, task-based nature of our recordings versus the extended monitoring protocols (e.g., 24-hour) in prior studies,[36,37] which may capture different aspects of autonomic regulation over time.[16] Alternatively, normalization of autonomic indices during inpatient recovery may have attenuated group differences.[38] These results suggest that clinically relevant interoceptive alterations in AN may not be fully captured by conventional physiological metrics alone, underscoring the added value of integrated perceptual and modeling-based approaches.

3. Self-report findings

Self-report ratings revealed modality-specific alterations in AN. Perceived stomach sensation intensity increased with stimulation but did not differ by group, despite clear behavioral impairments in AN. This dissociation between objective and subjective measures is consistent with prior literature distinguishing different facets of interoception[39], and highlights the limitations of relying solely on subjective ratings to capture visceral processing abnormalities. Notably, most self-report differences in the present study emerged in non-GI domains, aligning with evidence that interoceptive alterations in AN are modality- and context-dependent and that self-report measures may be confounded by factors such as alexithymia.[40] Rather than positioning self-report as a limitation, these findings underscore the value of triangulating subjective ratings with behavioral and computational indices to provide a more nuanced and informative assessment of visceral processing abnormalities

4. Limitations of the study

This study has several limitations. First, the sample included only weight-restored females with restrictive AN, limiting generalizability to males, individuals with binge-purge presentations, and those who are acutely underweight. Second, all participants were recruited from a single inpatient program, which may reduce applicability to more heterogeneous outpatient or community samples. Third, diagnoses were established during routine clinical care rather than through research-administered structured diagnostic interviews. Although all AN diagnoses were made using DSM-5 criteria by board-certified psychiatrists or psychologists with extensive experience in eating disorder assessment and treatment, and informed by multidisciplinary team consensus, the absence of formal inter-rater reliability data may limit comparability with studies using standardized diagnostic interviews. Fourth, while the vibrating capsule offers advantages in scalability and non-invasiveness, it primarily targets gastric and proximal small intestinal mechanoreceptors, leaving other visceral modalities (e.g., chemosensory, hormonal) unaddressed. Additionally, physiological recordings were brief and task-bound, potentially missing slower-evolving autonomic

© 2026 Verdonk C et al. *JAMA Psychiatry*.

dynamics detectable with extended monitoring. Fifth, illness status at 6 months was dichotomized into full relapse versus not full relapse for longitudinal analyses. This approach was chosen to enhance statistical feasibility and ensure adequate power, while also prioritizing detection of clinically severe outcomes. Although this approach combines intermediate outcomes, full relapse represents a clinically distinct and actionable risk state. Identifying predictors of this state remains a key goal for relapse prevention and targeted intervention. Sixth, although the six-month follow-up allowed for relapse prediction, longer-term trajectories of remission and recurrence (e.g., 1-year and beyond[20]) remain unexplored. Finally, although dissociations between interoceptive accuracy and sensibility have been reported in several psychiatric disorders, the present findings reflect a different pattern: reduced gastrointestinal interoceptive accuracy alongside altered self-reported intensity in other bodily domains. Because these ratings reflect momentary perceptual salience rather than trait-level interoceptive beliefs, the pattern does not map cleanly onto the classic "accuracy vs sensibility" distinction. Despite these limitations, the integration of behavioral, neural, and computational approaches in this study provides a novel and mechanistically rich framework for understanding and potentially intervening upon disordered interoception in AN.

© 2026 Verdonk C et al. *JAMA Psychiatry*.

eReferences

1. Attia E, Walsh BT. Eating Disorders: A Review. *JAMA*. Apr 8 2025;333(14):1242–1252. doi:10.1001/jama.2025.0132

2. Staller K, Abber SR, Burton Murray H. The intersection between eating disorders and gastrointestinal disorders: a narrative review and practical guide. *Lancet Gastroenterol Hepatol*. Jun 2023;8(6):565–578. doi:10.1016/S2468-1253(22)00351-X

3. Mayeli A, Al Zoubi O, White EJ, et al. Parieto-occipital ERP indicators of gut mechanosensation in humans. *Nature Communications*. 2023/06/13 2023;14(1):3398. doi:10.1038/s41467-023-39058-4

4. Ron Y, Halpern Z, Safadi R, Dickman R, Dekel R, Sperber AD. Safety and efficacy of the vibrating capsule, an innovative non-pharmacological treatment modality for chronic constipation. *Neurogastroenterology & Motility*. 2015;27(1):99–104.

5. Nelson AD, Camilleri M, Acosta A, et al. A single-center, prospective, double-blind, sham-controlled, randomized study of the effect of a vibrating capsule on colonic transit in patients with chronic constipation. *Neurogastroenterology & Motility*. 2017;29(7):e13034.

6. Rao SSC, Lembo A, Chey WD, Friedenberg K, Quigley EMM. Effects of the vibrating capsule on colonic circadian rhythm and bowel symptoms in chronic idiopathic constipation. *Neurogastroenterology & Motility*. 2020;32(11):e13890.

7. Grier JB. Nonparametric indexes for sensitivity and bias: computing formulas. *Psychological Bulletin*. 1971;75(6):424.

8. Stanislaw H, Todorov N. Calculation of signal detection theory measures. *Behavioral Research Methods*. 1999;31(1):137–149.

9. Friston K, FitzGerald T, Rigoli F, Schwartenbeck P, Pezzulo G. Active inference: a process theory. *Neural computation*. 2017;29(1):1–49.

10. Friston KJ, Parr T, de Vries B. The graphical brain: belief propagation and active inference. *Network Neuroscience*. 2017;1(4):381–414.

11. Smith R, Friston KJ, Whyte CJ. A step-by-step tutorial on active inference and its application to empirical data. *Journal of mathematical psychology*. 2022;107:1–60.

12. Smith R, Mayeli A, Taylor S, Al Zoubi O, Naegele J, Khalsa SS. Gut inference: A computational modelling approach. *Biological psychology*. 2021;164:108152.

13. Friston K, Mattout J, Trujillo-Barreto N, Ashburner J, Penny W. Variational free energy and the Laplace approximation. *NeuroImage*. 2007/01/01/ 2007;34(1):220–234. doi:10.1016/j.neuroimage.2006.08.035

14. Oostenveld R, Fries P, Maris E, Schoffelen JM. FieldTrip: Open source software for advanced analysis of MEG, EEG, and invasive electrophysiological data. *Computational intelligence and neuroscience*. 2011;2011:156869. doi:10.1155/2011/156869

15. Wolpert N, Rebollo I, Tallon-Baudry C. Electrogastrography for psychophysiological research: Practical considerations, analysis pipeline, and normative data in a large sample. *Psychophysiology*. 2020;57(9):e13599.

16. Laborde S, Mosley E, Thayer JF. Heart rate variability and cardiac vagal tone in psychophysiological research–recommendations for experiment planning, data analysis, and data reporting. *Frontiers in psychology*. 2017;8:213.

© 2026 Verdonk C et al. *JAMA Psychiatry*.

17. Bach DR. A head-to-head comparison of SCRalyze and Ledalab, two model-based methods for skin conductance analysis. *Biological psychology*. 2014;103:63–68.

18. Benedek M, Kaernbach C. Decomposition of skin conductance data by means of nonnegative deconvolution. *Psychophysiology*. 2010;47(4):647–658.

19. Benedek M, Kaernbach C. A continuous measure of phasic electrodermal activity. *Journal of neuroscience methods*. 2010;190(1):80–91.

20. Khalsa SS, Portnoff LC, McCurdy-McKinnon D, Feusner JD. What happens after treatment? A systematic review of relapse, remission, and recovery in anorexia nervosa. *Journal of eating disorders*. 2017;5:20. doi:10.1186/s40337-017-0145-3

21. Garner DM, Olmstead MP, Polivy J. Development and validation of a multidimensional eating disorder inventory for anorexia nervosa and bulimia. *International Journal of Eating Disorders*. 1983;2(2):15–34.

22. Fairburn CG, Beglin SJ. Eating disorder examination questionnaire. In: Fairburn CG, ed. *Cognitive behavior therapy and eating disorders*. Guilford Press; 2008:309–313.

23. Cooper PJ, Taylor MJ, Cooper Z, Fairbum CG. The development and validation of the Body Shape Questionnaire. *International Journal of eating disorders*. 1987;6(4):485–494.

24. Terry A, Szabo A, Griffiths M. The exercise addiction inventory: A new brief screening tool. *Addiction Research & Theory*. 2004;12(5):489–499.

25. Satterthwaite FE. Synthesis of variance. *Psychometrika*. 1941;6(5):309–316.

26. Luke SG. Evaluating significance in linear mixed-effects models in R. *Behavior research methods*. 2017;49:1494–1502.

27. Kenward MG, Roger JH. Small sample inference for fixed effects from restricted maximum likelihood. *Biometrics*. 1997:983–997.

28. *R: A Language and Environment for Statistical Computing*. R Foundation for Statistical Computing; 2024. https://www.R-project.org/

29. Bates D, Mächler M, Bolker B, Walker S. Fitting Linear Mixed-Effects Models Using lme4. *Journal of Statistical Software*. 10/07 2015;67(1):1 – 48. doi:10.18637/jss.v067.i01

30. Kuznetsova A, Brockhoff PB, Christensen RHB. lmerTest Package: Tests in Linear Mixed Effects Models. *Journal of Statistical Software*. 12/06 2017;82(13):1 – 26. doi:10.18637/jss.v082.i13

31. Maris E. Statistical testing in electrophysiological studies. *Psychophysiology*. Apr 2012;49(4):549–65. doi:10.1111/j.1469-8986.2011.01320.x

32. Maris E, Oostenveld R. Nonparametric statistical testing of EEG- and MEG-data. *Journal of neuroscience methods*. Aug 15 2007;164(1):177–90. doi:10.1016/j.jneumeth.2007.03.024

33. Pernet CR, Latinus M, Nichols TE, Rousselet GA. Cluster-based computational methods for mass univariate analyses of event-related brain potentials/fields: A simulation study. *Journal of neuroscience methods*. 2015;250:85–93.

34. Berg KC, Peterson CB, Frazier P, Crow SJ. Psychometric evaluation of the eating disorder examination and eating disorder examination-questionnaire: a systematic review of the literature. *The International journal of eating disorders*. Apr 2012;45(3):428–38. doi:10.1002/eat.20931

© 2026 Verdonk C et al. *JAMA Psychiatry*.

35. Gelman A. Scaling regression inputs by dividing by two standard deviations. *Statistics in medicine*. 2008;27(15):2865–2873.

36. Jenkins ZM, Eikelis N, Phillipou A, Castle DJ, Wilding HE, Lambert EA. Autonomic nervous system function in anorexia nervosa: a systematic review. *Frontiers in neuroscience*. 2021;15:682208.

37. Mazurak N, Enck P, Muth E, Teufel M, Zipfel S. Heart rate variability as a measure of cardiac autonomic function in anorexia nervosa: a review of the literature. *European eating disorders review*. 2011;19(2):87–99.

38. Mont L, Castro J, Herreros B, et al. Reversibility of cardiac abnormalities in adolescents with anorexia nervosa after weight recovery. *Journal of the American Academy of Child & Adolescent Psychiatry*. 2003;42(7):808–813.

39. Garfinkel SN, Seth AK, Barrett AB, Suzuki K, Critchley HD. Knowing your own heart: distinguishing interoceptive accuracy from interoceptive awareness. *Biological Psychology*. Jan 2015;104:65–74. doi:https://doi.org/10.1016/j.biopsycho.2014.11.004

40. Khalsa SS, Verdonk C. Interoception and Mental Health. In: Murphy J, Brewer R, eds. *Interoception: A Comprehensive Guide*. Springer; 2024:265–316:chap 9.

© 2026 Verdonk C et al. *JAMA Psychiatry*.
