# Supplementary material for: Gastrointestinal Interoception and Relapse in Anorexia Nervosa
Source: JAMA Psychiatry. 2026 Jun 17:e261301. Online ahead of print. doi: 10.1001/jamapsychiatry.2026.1301 (PMC13276667; doi:10.1001/jamapsychiatry.2026.1301)
Supplement: Supplement 3. — Data sharing statement [file jamapsychiatry-e261301-s003.pdf]

Data Sharing Statement

Verdonk. Gastrointestinal Interoception and Relapse in Anorexia Nervosa. *JAMA Psychiatry*. Published June 17, 2026. doi:10.1001/jamapsychiatry.2026.1301

Data
Additional Information: NCT05111977
Data available: No

Additional Information
Explanation for why data not available: The data are available via the NIH Data Archive (NDA).
